# Estimated Impacts of Prescribed Fires on Air Quality and Premature Deaths in Georgia and Surrounding Areas in the US, 2015–2020

**DOI:** 10.1021/acs.est.4c00890

**Published:** 2024-06-29

**Authors:** Kamal J. Maji, Zongrun Li, Ambarish Vaidyanathan, Yongtao Hu, Jennifer D. Stowell, Chad Milando, Gregory Wellenius, Patrick L. Kinney, Armistead G. Russell, M. Talat Odman

**Affiliations:** † School of Civil and Environmental Engineering, Georgia Institute of Technology, Atlanta, Georgia 30332, United States; ‡ National Center for Environmental Health, Centers for Disease Control and Prevention, Atlanta, Georgia 30329, United States; § School of Public Health, Boston University, Boston, Massachusetts 02118, United States

**Keywords:** prescribed burn, chemical transport model, air pollution, premature deaths

## Abstract

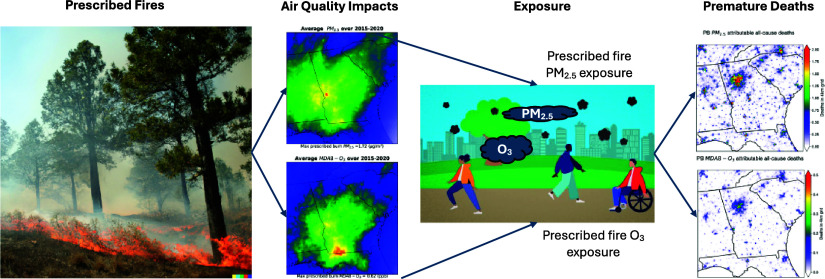

Smoke from wildfires poses a substantial threat to health in communities near and far. To mitigate the extent and potential damage of wildfires, prescribed burning techniques are commonly employed as land management tools; however, they introduce their own smoke-related risks. This study investigates the impact of prescribed fires on daily average PM_2.5_ and maximum daily 8-h averaged O_3_ (MDA8-O_3_) concentrations and estimates premature deaths associated with short-term exposure to prescribed fire PM_2.5_ and MDA8-O_3_ in Georgia and surrounding areas of the Southeastern US from 2015 to 2020. Our findings indicate that over the study domain, prescribed fire contributes to average daily PM_2.5_ by 0.94 ± 1.45 μg/m^3^ (mean ± standard deviation), accounting for 14.0% of year-round ambient PM_2.5_. Higher average daily contributions were predicted during the extensive burning season (January–April): 1.43 ± 1.97 μg/m^3^ (20.0% of ambient PM_2.5_). Additionally, prescribed burning is also responsible for an annual average increase of 0.36 ± 0.61 ppb in MDA8-O_3_ (approximately 0.8% of ambient MDA8-O_3_) and 1.3% (0.62 ± 0.88 ppb) during the extensive burning season. We estimate that short-term exposure to prescribed fire PM_2.5_ and MDA8-O_3_ could have caused 2665 (95% confidence interval (CI): 2249–3080) and 233 (95% CI: 148–317) excess deaths, respectively. These results suggest that smoke from prescribed burns increases the mortality. However, refraining from such burns may escalate the risk of wildfires; therefore, the trade-offs between the health impacts of wildfires and prescribed fires, including morbidity, need to be taken into consideration in future studies.

## Abstract 2

This study quantifies the adverse impacts of prescribed fires in Georgia and surrounding areas on ambient levels of PM_2.5_, ozone, and associated premature mortality.

## 1 Introduction

In recent years, escalating impacts of climate change have led to unprecedented levels of smoke exposure caused by wildfires across the globe. Within the United States (US), the annual acreage consumed by wildfires has doubled over the past two decades.^1^ Prescribed fires serve as a strategic land management tool used in reducing the buildup of combustible materials, or fuels, thereby lowering the risk of catastrophic wildfires, as well as in ecosystem restoration and habitat enhancement.^2−4^ Prescribed fires are carefully executed under specific environmental conditions.^5−7^ They are low-intensity fires, and the smoke they emit differs substantially from wildfire smoke in terms of constituents, concentrations, and heat release.^1^ Furthermore, prescribed burns are conducted on a regular basis (every two years or so) with smaller burned areas such that exposures to smoke plumes from prescribed fires are generally shorter in duration but occur more frequently than wildfire events.^4^

Over the span of 1985–2020, the annual average of prescribed burning in the US amounted to 11 million acres (about half the area of Kentucky).^1^ However, prescribed burns constitute only about 10% of the total treatments implemented by the US Forest Service. Currently, suppression remains the primary approach to wildfire management, though increased use of prescribed burns is planned.^5^ Of the total prescribed burning in the US, 71% of the burns (by number) are in the Southeast with a rate of increase of 0.15 million acres/year. Prescribed burning is responsible for ∼24% of the primary PM_2.5_ (particulate matter with aerodynamic diameter ≤2.5 μm) emissions in the Southeastern US.^1,6^ It is estimated that the prescribed fires contribute annually ∼10 to 15% of ambient PM_2.5_ to ∼20 to 30% during the extensive burning season (January–April) in the Southeastern US.^7−11^

As a dominating source of outdoor air pollution, prescribed burns are not without their own set of potential health risks or adverse health impacts.^12^ While more is known about the potential effects of wildfire smoke exposure,^13,14^ less is known about potential health threats from prescribed burns, especially for vulnerable populations. Afrin and Garcia-Menendez^15^ reported 70 excess mortality cases among older adults attributed to prescribed smoke PM_2.5_ exposure during the burning season in Georgia. Carter et al.^16^ estimated that human-ignited fire smoke was responsible for 7400 premature deaths in the US in 2003, which increased to 20 000 in 2018. Moreover, there are some indications of an uneven distribution of the burden. For example, Johnson-Gaither et al.^17^ investigated the susceptibility of African Americans to prescribed fire smoke exposure in Georgia and observed that permitted burns with the highest impact on air quality also corresponded to areas with higher African American populations.

While much of the research has primarily focused on prescribed burns during the extensive burning season, which accounts for approximately 60% of total burns in the Southeastern US, it is crucial not to disregard the impact of the out-of-season burns occurring during other times of the year due to their potential public health impacts. In addition, there is a scarcity of studies that have explored the influence of prescribed fire smoke contribution to ozone (O_3_). Given these considerations, there is a compelling need to comprehensively assess the year-round impacts of prescribed fire. This involves quantifying the contribution of prescribed burning to air pollution, including PM_2.5_ and O_3_ concentrations, to gauge the health burden associated with prescribed burn smoke exposure in the region.

Accordingly, here, we simulate the impacts of prescribed burns on daily 24-h average PM_2.5_ and maximum daily average 8-h O_3_ (MDA8-O_3_) during the 2015–2020 period in a portion of the Southeastern US that includes the entire state of Georgia and portions of surrounding states (i.e., Alabama, Florida, North Carolina, South Carolina, and Tennessee) and use those results to estimate the annual number of premature deaths attributable to short-term prescribed fire smoke exposure. We apply the following framework: (a) identify daily prescribed fire information from satellite-derived product Fire INventory from NCAR (FINN) (version 2.5) to estimate three-dimensional prescribed burning emissions for the Community Multiscale Air Quality (CMAQ) model (spatial resolution of 4 × 4 km^2^); (b) simulate the prescribed fire contributions to daily average PM_2.5_ and MDA8-O_3_ using CMAQ; (c) fuse simulated PM_2.5_ and MDA8-O_3_ fields with daily observations at ambient surface monitors to generate an “observation-adjusted prescribed burn impact”; and (d) use the “observation-adjusted prescribed burn impact” data to assess premature deaths resulting from short-term exposure to PM_2.5_ and MDA8-O_3_. A simplified flowchart of the study framework is presented in Figure S1.

## 2 Materials and Methods

### 2.1 Prescribed Burn Identification and Emissions

Prescribed burns are the major source of PM_2.5_ pollution in the Southeastern US; however, prescribed fire activity information (e.g., location, date, time, and burned area) in burn permit records may be inaccurate and those records are not always readily accessible for all states.^6^ Satellite-based remote sensing products can fill data gaps; however, they do not differentiate prescribed burns from other wildland fires. In the present study, we follow the method developed by Li et al.^18^ to identify prescribed burns in the FINN database. Similar to clustering algorithms developed for detecting large wildfires,^19,20^ this method aggregates the FINN fires based on spatial and temporal separation. First, we removed agricultural burns by considering fires that occurred in agriculture lands. Then, we focused on detecting large wildfires. Prescribed burns typically start and end on the same day, while wildfires can last multiple days, so we assumed that the fires that have more than 1 day duration are wildfires. In this way, we matched about 20% of the Wildland Fire Interagency Geospatial Services (WFIGS) wildfire records.^19,20^ The permit-reported burned areas are considered more accurate than the satellite-reported burned areas;^19^ therefore, a linear regression model was used to calibrate the FINN-based prescribed burned area with area in permit records at 4-km resolution spatial grid level. The resulting adjusted burned area is used as an input to the BlueSky Smoke Modeling Framework to estimate three-dimensional hourly prescribed burning emissions for CMAQ.^20−22^ BlueSky links together fire location, fire size, fire type, fuel loading, fuel consumption, speciated emissions, smoke dispersion, and plume trajectories. Three-dimensional gridded meteorological data required for trajectory and dispersion calculations are provided by the Weather Research and Forecasting (WRF) model.^23−25^ The daily total emissions from FINN and BlueSky were highly correlated and consistent with each other.^18^ However, we chose to use the fire emissions calculated by using BlueSky rather than directly using the fire emissions provided by FINN because BlueSky has a more advanced fuel classification algorithm than FINN.^26^ It also contains a comprehensive archive of emission factors for these fuels.^27^

### 2.2 Air Quality Simulations

We simulated daily air quality from 1st January 2015 to 31st December 2020 using meteorology from the WRF model in CMAQ version 5.2,^28^ a fully coupled chemical transport model (CTM). CMAQ employs state-of-the-science representations of atmospheric processes affecting transport, transformation, and deposition of pollutant species.^28−30^ Emissions include anthropogenic emissions based on the National Emission Inventory (NEI)^31^ as well as biogenic, windblow dust, and wildfire emission,^32^ while prescribed fire emissions were derived from the BlueSky Smoke Modeling Framework, as described in Section 2.1.^18^ We used the EPA 2011v6 Platform^33^ for modeling the anthropogenic emissions. The sectorized inventories used were the 2011 NEI, projected to 2017, and kept the same for 2015–2020. The modeling domain covers Georgia and surrounding areas of the Southeastern US from 28.98°N and −87.82°W to 36.28°N and −79.13°W with 180 × 180 grid boxes at 4 km × 4 km horizontal resolution (Figure S2), with 35 vertical levels extending up to 50 hPa. The concentrations of pollutants were calculated by simulating two scenarios with CMAQ, a baseline simulation with all emissions (*C*_all_^s^), and a second simulation in which prescribed fire emissions were not included (*C*_no–PB_^s^). This allowed to quantify the impact from prescribed burns as
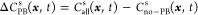
1where Δ*C*_PB_^s^ is the concentration associated with prescribed burning emissions, and ***x*** and *t* indicate the variation in three-dimensional space and time.

### 2.3 Data-Fusion Method

The CMAQ, like any other CTM, has uncertainties related to emissions inputs, meteorological parameter data, and physical/chemical transport processes; hence, the simulation results differ from the results of field measurements.^34,35^ To reduce the model biases and error, in the current study, we calibrated daily average PM_2.5_ and MDA8-O_3_ results simulated by CMAQ with observation, following the data-fusion (DF) approach of Friberg et al.^34^ For data-fusion and model evaluation, observed daily ambient PM_2.5_ and MDA8-O_3_ concentrations were obtained from, respectively, 99 and 105 EPA-AQS (Environmental Protection Agency-Air Quality System) monitors in the study area.^36^Equation 2 reflects the regression model applied to produce optimized fused concentration fields *C*_all_^DF^(***x***, *t*) by computing a weighted average with the weight depending on the spatial autocorrelation of observations and the correlation between observations and CMAQ simulation results (S1.1):
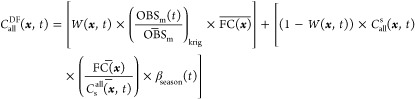
2where
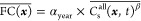

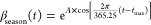


The resulting product *C*_all_^DF^(***x***, *t*) is a new data-fused field that captures the temporal variations in local observations as well as spatial variability in CMAQ simulations. Here, OBS_m_ is daily observations at each monitor (m), overbar indicates temporal averaging (annual), α_year_ is a regression parameter derived for each year, β is a parameter derived for all years, and *W* is an average weighting factor for the study period. β_season_ is the seasonal correction function modeled as a smooth trigonometric function with two fitted parameters, amplitude (*A*) and day of peak correction (*t*_max_).

In locations without a monitor, the fused fields relied more heavily on CMAQ simulations. This method provided a way to extend the coverage of air quality assessments to areas where direct observational data were unavailable, thereby improving the understanding of spatial and temporal variability of air pollution across a broader area.

The fused daily total PM_2.5_ and MDA8-O_3_ fields were multiplied by the ratio of the burn impacts to the total PM_2.5_ and MDA8-O_3_ from CMAQ for each day and each grid cell to generate an ‘observation-adjusted burn impact’, Δ*C*_PB_^DF^(***x***, *t*), as follows:
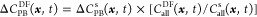
3

### 2.4 Mortality Impact Assessment

We estimated prescribed burn smoke-associated premature deaths using the log–linear association of concentration–response functions (CRFs) for premature mortality impacts from acute exposures. In this study, we estimated all-cause, cardiovascular, and respiratory premature deaths attributed to daily average PM_2.5_ and MDA8-O_3_ exposure. General forms of CRFs used to calculate prescribed burn smoke exposure-attributable mortality are provided below:^14,37−40^
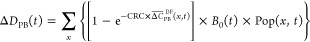
4where Δ*D*_PB_(*t*) is the cause-specific excess premature deaths due to prescribed burn smoke exposure for a year *t*; Δ*C*_PB_^DF^(*x*, *t*) is the county-level annual average air pollution contributed by prescribed burns, obtained after regridding the pollution concentration from eq 3 at county-level; CRC is the concentration response (CR)-coefficient, *B*_0_(*t*) is the county-specific and cause-specific baseline incidence rates; and Pop(*x*, *t*) is the county-level exposed population. *B*_o_(*t*) × Pop(*x*, *t*) is the cause-specific mortality registered in the county. The county-level registered mortality data were obtained from the Centers for Disease Control and Prevention’s (CDC’s) National Vital Statistics System. The county-level results were aggregated to obtain summaries for Georgia and the study domain.

For O_3_, the associated mortality impacts were determined by its concentration.^41^ However, the mortality impacts of PM_2.5_ are related to its composition.^42,43^ Previous studies estimated premature deaths for prescribed burn PM_2.5_ using CR-coefficients developed from all-source-specific total PM_2.5_ mass.^16,44^ However, Aguilera et al.^45^ found that risks associated with wildland fire smoke are higher compared to emissions from other sources like industries and power generation.^45^ They reported that exposure to wildfire smoke could lead to a 10-fold increase in the risk of respiratory hospitalizations, relative to other PM_2.5_ sources, which may lie, in part, with high contents of black carbon (BC) and organic carbon (OC) and high aromaticity of wildfire-PM_2.5_. To calculate the mortality attributable to prescribed fire PM_2.5_, we used the CR-coefficient reported by Chen et al.^13^ from a pooled meta-analysis (Table S1). These coefficients were developed to quantify the association between short-term exposure to wildfire-related PM_2.5_ and mortality.^13^ For short-term MDA8-O_3_ exposure-attributed mortality, the CR-coefficient is derived from a meta-analysis study and an epidemiological study conducted based on outdoor O_3_ exposure in the US (Table S1).^46,47^

As a comparative analysis, we employed the same mortality assessment method, utilizing gridded pollution and gridded population data along with state-specific baseline mortality from Global Burden of Disease (GBD, 2019)^48^ to estimate prescribed burn-attributable premature deaths. Population data at a resolution of 1 × 1 km^2^ from 2015 to 2020 were obtained from the Gridded Population of the World (GPW) (https://www.worldpop.org/) and were resampled using the nearest neighbor approach to match the CMAQ grid.

## 3 Results and Discussions

**Figure 1:**
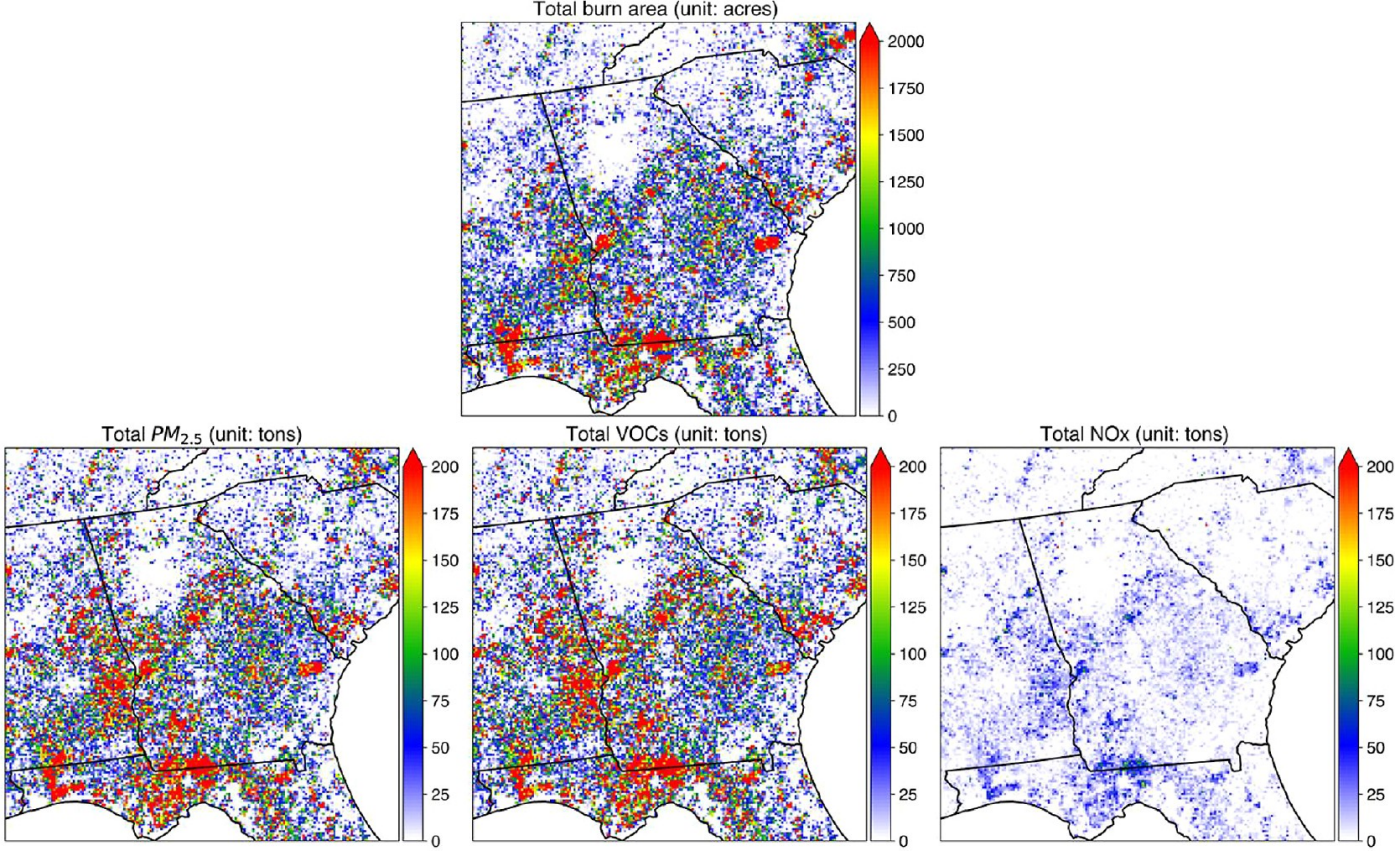
Spatial distribution of the total prescribed burned area observed by adjusted-FINN (top) and corresponding emission of total PM_2.5_, VOCs, and NOx (bottom) during 2015–2020 (units are acres for burn area and tons for PM_2.5_, VOCs, and NOx).

The clustering algorithm estimated nearly 70% of total fires as prescribed fire, covering 13.3 million acres of the study domain during the study period. The highest burn area was observed in 2017 with 2.74 million acres of prescribed burns (Figure 1). On average, 68% of the total prescribed burns are conducted during the burning season. The largest amounts of prescribed burning were recorded in Southwest Georgia. In these six years, prescribed burning contributed an estimated 1.7 million tons of PM_2.5_, 0.19 million tons of nitrogen oxides (NOx), and 1.76 million tons of volatile organic compound (VOC) emissions. The highest prescribed burn-related pollutant emissions were observed in 2017 (PM_2.5_: 0.35 million tons; NOx: 0.04 million tons; VOCs: 0.37 million tons; Figures S3–S6 and Table S2).

### 3.1 Model Performance Evaluation

**Figure 2:**
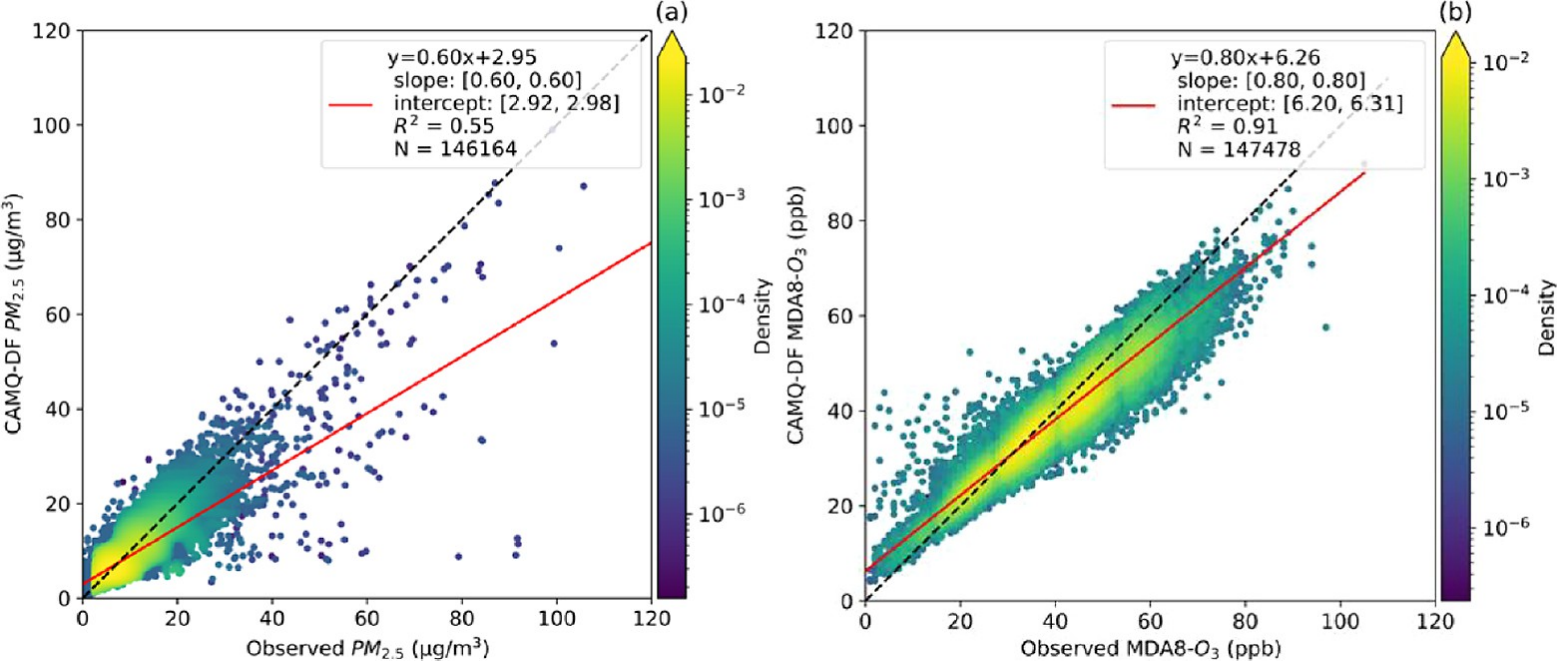
Density scatterplots of observed and data-fusion (DF) concentrations of (a) daily average PM_2.5_ and (b) MDA8-O_3_ during 2015–2020. The dotted line shows 1:1.

Evaluation of CMAQ indicated that the model generally underestimated PM_2.5_ (by ∼20%) and overestimated MDA8-O_3_ (by ∼40%) throughout the study period. After data-fusion, the model performance improved both for daily PM_2.5_ and MDA8-O_3_. The data-fused fields overestimate PM_2.5_ by ∼0.6% and slightly higher (by ∼2%) in the burning season. The fusion method captures the observed PM_2.5_ reasonably well at all monitoring stations (coefficient of determination (*R*^2^) = 0.55, mean bias (MB) = −0.39 μg/m^3^, root-mean-square error (RMSE) = 3.49 μg/m^3^, and normalized mean bias (NMB) = −4.69%) (Figure 2a). During the extensive burning season, both observed and simulated PM_2.5_ concentrations were higher than during any other season. During that period, the simulation captures the observed PM_2.5_ more accurately (*R*^2^ = 0.71, MB = −0.39 μg/m^3^, RMSE = 2.28 μg/m^3^, and NMB = −4.75%) as compared to the low-burn season (May–September) (*R*^2^ = 0.37, MB = −0.34 μg/m^3^, RMSE = 4.64 μg/m^3^, and NMB = −3.87%).

Data-fusion provided good agreement with the USEPA measurements for MDA8-O_3_, with *R*^2^ = 0.91 and RMSE = 4.03 ppb (MB = −1.73 ppb and NMB = −4.38%) (Figure 2b), and the model performance is similar during the extensive burning season (*R*^2^ = 0.89, MB = −1.51 ppb, RMSE = 3.67 ppb, and NMB = −3.64%) and low-burn season (*R*^2^ = 0.91, MB = −2.03 ppb, RMSE = 4.35 ppb, and NMB = −5.06%) (Table S3 and Figure S7).

### 3.2 Cross-Validation Performance

The data-fusion method performance was evaluated using a comprehensive 10-fold 10% data withholding cross-validation (CV) analysis. Across 99 and 105 monitors for PM_2.5_ and MDA8-O_3_, respectively, over six years, the number of withheld data corresponding to the number of observations was 146 thousand for PM_2.5_ and 147 thousand for MDA8-O_3_. The data withholding data-fusion results (Table S4) also had small MB, RMSE, and NMB and larger *R*^2^ values compared to the CMAQ results. The average of all 10 CV results meets the criteria and goals recommended by Emery et al.^49^ for air quality modeling, both for MDA8-O_3_ (*R*^2^ = 0.89, MB = −1.73 ppb, RMSE = 4.18 ppb, and NMB = −4.36%) and PM_2.5_ (*R*^2^ = 0.54, MB = −0.39 μg/m^3^, RMSE = 3.53 μg/m^3^, and NMB = −4.69%).

The data-fusion method was also evaluated using a leave-one-location-out cross-validation (LOLO CV) procedure, which was implemented to account for the spatial and temporal dependence of the data.^50^ This approach aimed to provide more realistic estimates of the prediction error. However, we observed that the average results from all LOLO CV evaluations for PM_2.5_ (*R*^2^ = 0.55, MB = −39 μg/m^3^, RMSE = 3.47 μg/m^3^, and NMB = −4.65%) and MDA8-O_3_ (*R*^2^ = 0.90, MB = −1.73 ppb, RMSE = 4.03 ppb, and NMB = −4.38%) were nearly identical to those obtained from 10-fold CV (Table S4). This observation diverges from findings in other studies, which often report significant differences between these two methods.^51^ The unexpected similarity in the results may be due to the specific characteristics of the data set and data-fusion approach. It is important to note that the LOLO CV entails a significant computational cost. In our analysis, with around 100 monitoring locations, there were essentially 100 folds in LOLO CV. This translates to a computational burden roughly 10 times greater than that of a standard 10-fold CV. Di et al.^52^ demonstrated that using spatial or temporal folds can provide a more efficient estimate of the prediction error in air pollution models due to spatial and temporal dependencies in the data.

Limited observational data in smoke-impacted areas may limit the accuracy of data-fused prescribed fire impact estimates. Such a lack of observations can be alleviated, in part, by using low-cost sensors; however, the performance of such sensors is still questionable when applying them to detect impacts from prescribed fires in high-concentration environments.^53^

### 3.3 Impacts of Prescribed Burns on Air Quality

**Figure 3:**
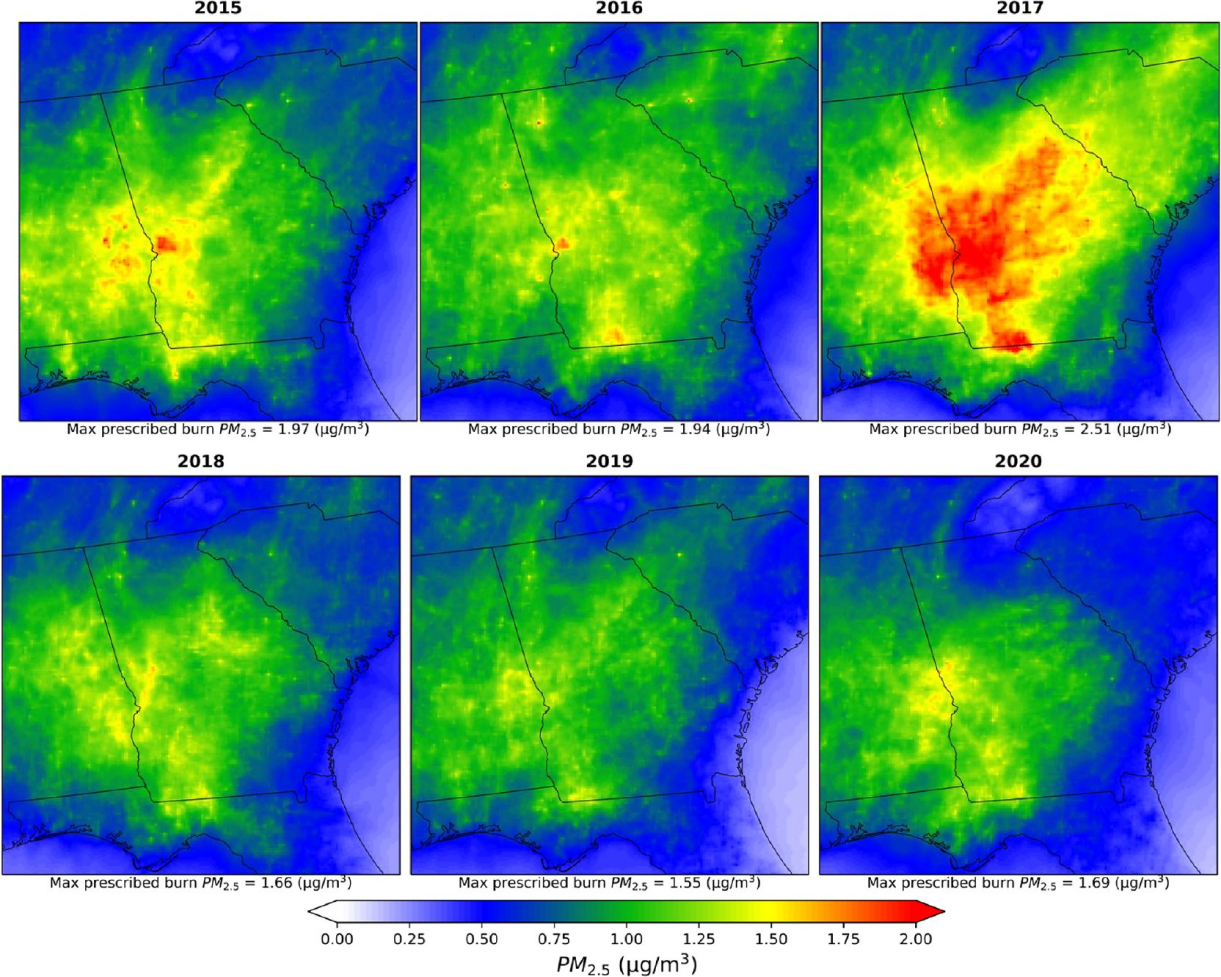
Spatial distributions of yearly average prescribed burn specific PM_2.5_ concentrations (μg/m^3^) during 2015–2020.

**Figure 4:**
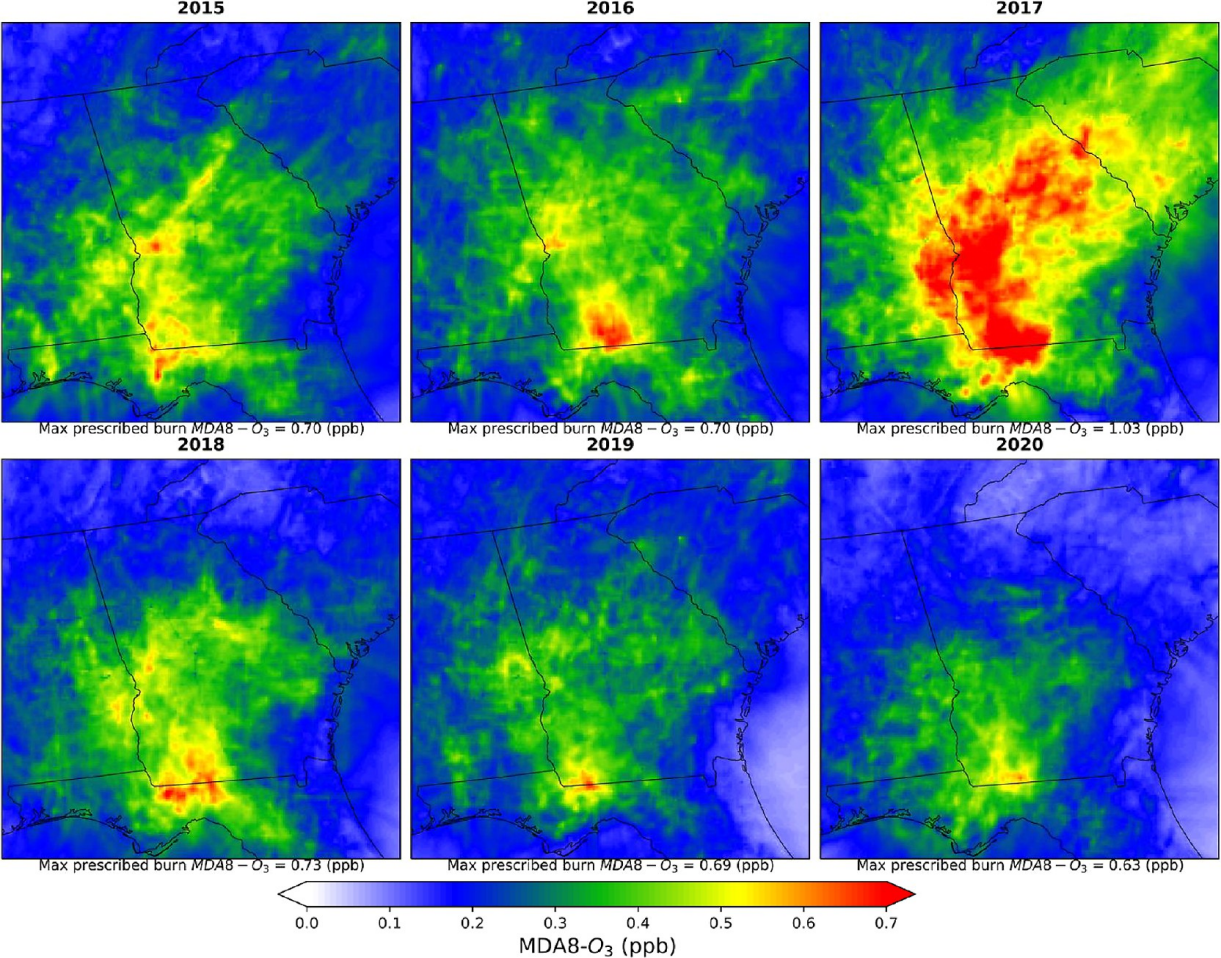
Spatial distributions of yearly average prescribed burn specific MDA8-O_3_ concentrations (ppb) during 2015–2020.

**Figure 5:**
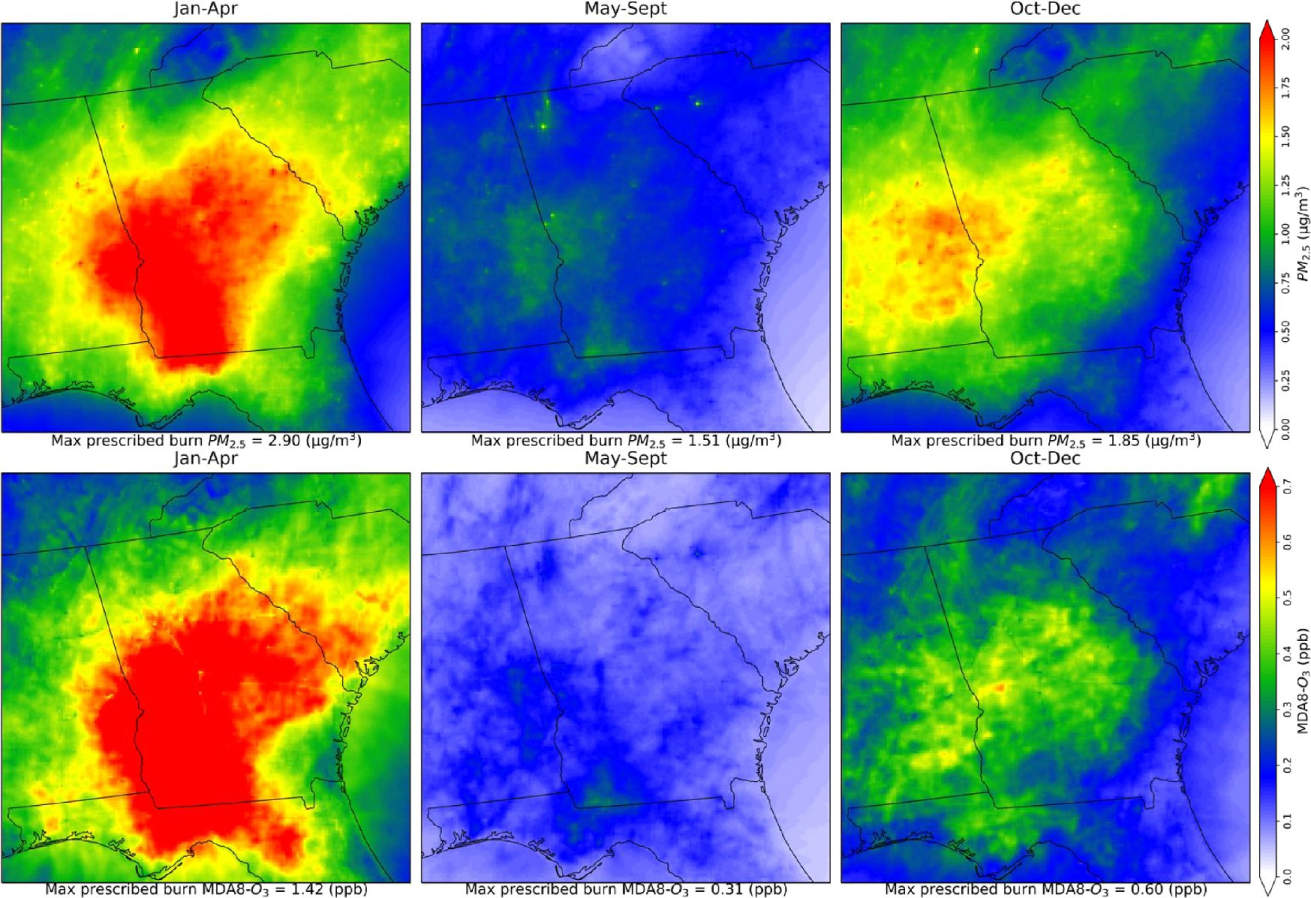
Spatial distributions of seasonal average prescribed burn specific PM_2.5_ (μg/m^3^) (top row) and MDA8-O_3_ concentrations (ppb) (bottom row) during 2015–2020. January–April is the extensive burning season, May–September is the low-burning season, and October–December is the cold season.

The spatial distributions of 2015–2020 annual mean PM_2.5_ and MDA8-O_3_ concentrations resulting from prescribed fires are shown in Figures 3 and 4, respectively. Over 2015–2020, prescribed burns contributed 0.94 ± 1.45 μg/m^3^ (mean ± standard deviation (SD), where SD is based on the variation of impacts in different grid cells) (median (MD): 0.41 μg/m^3^) to the daily average PM_2.5_ [range: 0.0–14.3 μg/m^3^] across the domain, which is, on average, around 14% of the ambient PM_2.5_. The lowest annual average burn impacts were observed in 2019: 0.80 ± 1.29 μg/m^3^ (MD: 0.36 μg/m^3^) and the highest in 2017: 1.28 ± 2.10 μg/m^3^ (MD: 0.40 μg/m^3^) (Table S5). The counties near the central western border of Georgia with Alabama are highly impacted by prescribed fires (e.g., Chattahoochee, Muscogee in Georgia, and Russell County in Alabama), experiencing a contribution to daily average PM_2.5_ of 1.60 ± 3.20 μg/m^3^ over 2015–2020. The highest prescribed burn impacts were observed during the extensive burning season. During this season, prescribed burns contribute an average of 20% to total atmospheric PM_2.5_ [average: 1.43 ± 1.97 μg/m^3^; MD: 0.67 μg/m^3^] (Figure 5).

Within Georgia, prescribed burns contributed 1.08 ± 1.54 μg/m^3^ to daily average PM_2.5_ (∼17% of the ambient PM_2.5_). The annual mean contribution ranged from 0.93 ± 1.20 μg/m^3^ in 2020 to 1.39 ± 1.94 μg/m^3^ in 2017, or 14–19% of ambient PM_2.5_ concentration in Georgia. In the extensive burning season, daily mean contribution ranged from 1.10 ± 1.29 to 2.40 ± 2.57 μg/m^3^, or 18–32% of total PM_2.5_ concentration (Table S6). Prescribed burning contributed above 70% of ambient PM_2.5_ in Georgia on the highest burned area days. For example, February 14 had the highest burn area in 2015 (29 910 acres) and consequently average PM_2.5_ was 9.36 μg/m^3^ or ∼72% of ambient PM_2.5_ was due to prescribed fire on that day (Figure S7). Prescribed burns have the potential to greatly increase PM_2.5_ concentrations often surpassing the National Ambient Air Quality Standard (NAAQS) (35 μg/m^3^) and reaching above hazardous levels (>250 μg/m^3^) in some grid cells. On 130 days during the study period, prescribed burns contributed 10% or more of the daily air quality standard for PM_2.5_ (≥3.5 μg/m^3^) to the average ambient PM_2.5_ concentration in Georgia.

As the prescribed burns are conducted under conditions of lower temperature and higher humidity compared to weather more conducive to wildfires, leading to lower NOx emissions, less O_3_ is formed as compared to wildfires.^6^ Prescribed fire was responsible for 0.36 ± 0.61 ppb (MD: 0.15 ppb) daily average increase of MDA8-O_3_ (range: 0–6.8 ppb) in the study domain, which is around 0.8% [range: 0–12.6%] of ambient MDA8-O_3_. The lowest annual average MDA8-O_3_ produced by prescribed fire was 0.30 ± 0.51 ppb (MD: 0.11 ppb) (0.8% of annual ambient MDA8-O_3_) in 2015, and the highest contribution was 0.52 ± 0.87 ppb (MD: 0.17 ppb) (1.0% of annual ambient MDA8-O_3_) in 2017. The counties near the central-southern border of Georgia with Florida (e.g., Thomas, Grady, and Leon County) are significantly affected by prescribed fires, with a daily average contribution of 0.70 ± 0.46 ppb to MDA8-O_3_. It is crucial to note that areas with elevated exposure to prescribed fire-related PM_2.5_ and MDA8-O_3_ are distinct. While high concentrations of smoke PM_2.5_ are observed near the burn location, the formation of O_3_ takes time, allowing emitted pollutants to stray from the source location in the interim. During the extensive burning season, prescribed fire contributed an average of 0.62 ± 0.88 ppb (MD: 0.28 ppb) or 1.3% to ambient MDA8-O_3_ (Figure 5).

Specifically, within Georgia, prescribed burning contributes an average of 0.37 ± 0.73 ppb (MD: 0.12 ppb) [range: 0–8.10 ppb] to MDA8-O_3_, which is around 1.0% [range: 0–25.2%]. During the extensive burning season, contribution to MDA8-O_3_ was 0.67 ± 1.06 ppb (MD: 0.26 ppb) or 1.8%. The highest prescribed fire contributions to MDA8-O_3_ were observed on the highest burned area days. For example, on February 14, 2015, prescribed burning contributed 3.4 ppb or 7.4% to MDA8-O_3_ (Figure S8). Prescribed fire smoke events can increase the MDA8-O_3_ values to above 150 ppb in some grid cells. During the study period, there are 270 days when Georgia experienced significant prescribed fire contribution to MDA8-O_3_ (>1% of ambient MDA8-O_3_ standard).

### 3.4 Impacts of Prescribed Fire on Mortality

The premature mortality estimated for prescribed fire-related exposure depends on several factors, including changes in air quality, population, and mortality over time. The population in our study domain was 30.6 million, and average population-weighted exposures to prescribed fire PM_2.5_ and MDA8-O_3_ were 0.97 μg/m^3^ and 0.36 ppb, respectively, over the domain. The highest population-weighted exposure occurred in 2017, with average exposures to prescribed fire PM_2.5_ and MDA8-O_3_ values of 1.32 μg/m^3^ and 0.52 ppb, respectively. In Georgia, the average population-weighted prescribed fire PM_2.5_ was 1.13 μg/m^3^ over 2015–2020.

The estimate of all-cause premature deaths attributed to short-term prescribed fire PM_2.5_ exposure was 2665 (95% confidence interval (CI): 2249–3080) from 2015 to 2020. Among all premature deaths, 26% were due to cardiovascular causes, and 10% were related to respiratory causes. Additionally, prescribed fire MDA8-O_3_ is associated with 233 (95% CI: 148–317) all-cause premature deaths across the entire study area, over 2015–2020. The highest number of all-cause premature deaths associated with prescribed fire-related PM_2.5_ and MDA8-O_3_ exposures was for 2017, with 511 (95% CI: 431–590) and 48 (95% CI: 31–65) premature deaths, respectively. These higher excess mortality numbers can be attributed to a larger acreage of land treated with prescribed burning that year, which, in turn, was associated with higher prescribed burn-related emissions. On average, there were a total of 200 (95% CI: 169–231) all-cause premature deaths (PM_2.5_- and MDA8-O_3_-attributale deaths) per million acres (∼0.4 million ha) of prescribed burn across the study domain.

Within Georgia, the estimated total premature deaths attributed to PM_2.5_ and MDA8-O_3_ exposures from prescribed burns were 930 (95% CI: 785–1074) and 83 (95% CI: 52–112), respectively, during the same period. Notably, Georgia accounted for 35% of the estimated prescribed fire-attributable total mortality in the study domain. However, when considering total deaths from all causes in Georgia during the study period, the number of premature deaths attributable to prescribed fire smoke exposure represented only ∼0.2% of the total mortality.^54^ The yearly premature deaths are reported in Table S11.

For comparative analysis, we utilized the same CR-coefficient, along with state-specific baseline mortality data from the GBD report and grided population data from GPW to assess all-cause premature deaths. Our estimation resulted in a total of 2915 (95% CI: 2459–3372) all-cause premature deaths attributable to short-term prescribed burn PM_2.5_ exposure and 271 (95% CI: 171–367) to short-term prescribed burn MDA8-O_3_ exposure, over 2015–2020 across the entire study domain. Whereas, within Georgia, the total estimated premature deaths attributed to prescribed fire-related PM_2.5_ and MDA8-O_3_ exposure were 1234 (95% CI: 1041–1426) and 99 (95% CI: 62–133), respectively, during 2015–2020. The instances of high premature all-cause deaths are situated in regions with higher population density, distinct from the areas with elevated concentrations of prescribed burn smoke (Figure S10). These estimated values are higher than county-level analysis, as county-specific cause-specific mortalities differ from the state averages, and regridding the county-level concentration data may underestimate the actual exposures.

### 3.5 Uncertainty and the Knowledge Gap

This study is unique in its combined use of the satellite observations for prescribed fire detection, its data-fusion method, and linkage to population exposures to gain new insights about prescribed fire impacts on air quality and associated premature deaths. The FINN product is developed based on satellite-detected thermal anomalies from vegetation fires.^55^ Indeed, one of the main disadvantages of all remote sensing thermal anomaly products is that they do not detect most of the fires less than approximately 100 ha and some understory fires, both of which can be a significant source of emissions to the atmosphere.^56,57^ Moreover, the satellites measured a 10–20% larger burn area compared to the actual burn area reported in ground-based prescribed burning databases, suggesting that they also include at least some wildfires.^58−60^ The burn area can be calibrated based on actual permit record data. However, burned areas obtained from permit records can be inaccurate; of note, there is ∼15% difference between recorded burn areas and actual burned areas.^19^ Other ground-based or complementary methods, such as drone-based measurement, may be necessary to ensure comprehensive prescribed burn area monitoring. One limitation of the algorithm used to discern prescribed fires from FINN is that if a wildfire was extinguishing in the same day it was detected, it is considered to be a prescribed fire. Therefore, some of the emissions calculated in this paper could be due to wildfires.

The prescribed burn area in the domain remains relatively constant interannually (2.2 ± 0.29 million acres/year), but the emissions are not necessarily proportional to the burned area. The fuel type and density, amount of fuel consumed, temperature, fuel moisture, and wind speed and direction are also drivers in determining the emissions. For example, PM_2.5_ and VOCs emissions per acre of prescribed burn were about 2.2 and 2.4%, respectively, higher in 2019 compared to 2015. Most of the previous studies have considered prescribed fire during the extensive burning season; however, there was significant off-season exposure. In the cold season (October–December), prescribed fire contributes to PM_2.5_ and MDA8-O_3_ in the study domain, on average, 1.13 ± 1.46 μg/m^3^ (MD: 0.58 μg/m^3^) (16% of ambient PM_2.5_) and 0.35 ± 0.47 ppb (MD: 0.17 ppb) (0.8% of ambient MDA8-O_3_), respectively. The summertime burn ban reduces the formation of ground-level O_3_ by prohibiting certain open burning activities from 1st May through 30th September in 54 counties out of 159 counties in Georgia.^61^ However, burning continues in the surrounding states. In summer, prescribed fire contributes an average of 0.59 ± 0.50 μg/m^3^ (MD: 0.42 μg/m^3^) to PM_2.5_ and 0.12 ± 0.10 ppb (MD: 0.10 ppb) to MDA8-O_3_ in Georgia. The prescribed burning in regions adjacent to the study domain should be considered, as it may affect the air quality within the domain and potentially influence the outcomes of the study.

When smoke plumes from fires mix into urban areas, they alter the local photochemical environment.^62^ As a result, urban O_3_ levels can be influenced by upwind O_3_ production from smoke as well as enhanced O_3_ production within the urban environment.^63,64^ Liu et al. found an increase of 12–30 ppb in MDA8-O_3_ during early spring prescribed burning in the urban Southeastern US.^65^ Local or regional O_3_ precursors from wildland fires can be advected into marine environments, which may then recirculate back into populated areas. Models and observations indicate that O_3_ can increase over water bodies due to inhibited deposition, shallower boundary layers, and ship emissions.^66,67^ We found similar behavior of O_3_ over the coastal region of Georgia (Figure S11), where prescribed burn O_3_ reached above 15 ppb, although the impact was depressed by marine halogens.^68^

Studies have suggested that compositional differences in wildland fire PM_2.5_ can be associated with higher health risks than typical urban PM_2.5_,^45^ raising even more concern for human health,^69−71^ with strong evidence that links short-term wildfire smoke exposures to increased all-cause mortality among older adults and infants.^72−74^ Recent epidemiological studies reported the association between wildfire (*sic*) smoke PM_2.5_ and mortality, using wildland fire PM_2.5_ exposure field at coarse resolution (≥10 × 10km^2^), which was generated primarily using a satellite fire product and a chemical transport model.^13,75^ Satellite fire products like the Global Fire Emissions Database (GFED) and the Hazard Mapping System (HMS) include all types of wildland fires and do not separate wildfire, prescribed fire, or agricultural fires.^76−78^ The coarse resolution^79^ of the wildfire-PM_2.5_ concentration field, including all types of fire emissions, may impact the association between wildfire-PM_2.5_ and deaths in epidemiological studies.

Wildfires, being uncontrolled events, may produce PM_2.5_ with a more varied chemical composition, potentially encompassing more harmful substances due to the diversity of materials consumed in the fire compared to prescribed fire-generated PM_2.5_.^6,80,81^ However, there is a notable absence of literature comparing the differential toxicity of PM_2.5_ from wildfires and prescribed fires, and no epidemiological studies have been conducted to evaluate the relationship between mortality and exposure to PM_2.5_ from prescribed fires. Therefore, applying a CR-coefficient for wildfire smoke exposure on prescribed fire smoke may under- or overestimate the excess premature deaths. Future research should aim to develop CR-coefficient specific to prescribed fire PM_2.5_ exposure to improve the accuracy of health impact assessments. Additionally, the burden of all-cause premature mortality attributed to short-term wildland smoke PM_2.5_ exposure was often estimated using all-source PM_2.5_-related CR-coefficients. For example, the USEPA used^82^ the all-source PM_2.5_-related CR-coefficient from the Zanobetti and Schwartz study to estimate premature deaths attributable to short-term wildfire and prescribed fire PM_2.5_ exposure.^83^ Using the CR-coefficient reported by Zanobetti and Schwartz, we estimated that the all-cause premature deaths attributable to short-term prescribed fire PM_2.5_ exposure in 2017 were 335 (95% CI: 257–416) over the study domain. In contrast, using a wildfire-specific CR-coefficient for PM_2.5_, our estimate was 646 (95% CI: 545–747) premature deaths, which is roughly twice as high (Table S11). Additionally, the links between prescribed fire PM_2.5_ and MDA8-O_3_ and various morbidities, such as asthma exacerbations and increased respiratory-related hospital admissions,^73,84^ were not addressed in this study.

The CMAQ-simulated surface pollution concentrations are highly influenced by the smoke plume rise or injection height used in air quality models. Plume rise is widely recognized as an area of uncertainty in smoke modeling.^85^ Traditionally, many plume rise models relied on Briggs plume rise equations, which were originally developed for industrial smokestacks.^86^ However, several studies suggest that this approach may not be appropriate for wildland fires^87,88^ and could lead to about 20% uncertainty in the final estimation of pollutant concentrations.^89^ Further research efforts are necessary to improve chemical properties of prescribed fire smoke, fuel consumption and emission factors to predict the burn impacts more precisely.^90,91^

## 4 Policy Implications and Future Work

Expanded prescribed fire activity in the US can be a valuable strategy to mitigate the risks of higher smoke exposure levels from wildfires. However, to ensure that prescribed burns do not have a detrimental impact on air quality or public health, it is essential to develop strategies that minimize adverse effects. The warming and drying climate, accumulation of fuels, and the expansion of the wildland–urban interface raise concerns about the health effects of wildland fire smoke on residents.^5,92,93^ The long-term emissions and air quality impacts of periodic prescribed burning are not well-quantified, and more research is needed in this area. Studies have shown that carbon emissions per hectare from prescribed burns over many decades are similar to or slightly higher than what would have been emitted by wildfires over the same period. However, prescribed burns tend to emit lower PM_2.5_ for a shorter duration compared to large wildfires.^94,95^

The overall wildland area to be treated by prescribed fire is likely to increase in the future to reduce the area burned in wildfires. This underscores the importance of efficient strategies for limiting exposure to prescribed fire smoke.^96−98^ At a minimum, the forecast and monitored air quality must be communicated to sensitive populations in a timely fashion. To facilitate future research, a centralized repository to store prescribed fire information can be developed for better accessibility of prescribed burn data. Such a repository would include, but not be limited to, information on location, timing, actual acres burned, fuel type and fuel loading information, and any air quality monitoring data collected, and this can enhance the overall effectiveness of the prescribed fire model and air quality management. The trade-offs between the air quality and health impacts of prescribed burning and wildfires are unknown; they should be evaluated and considered in policy-making together with the need for managing wildfires, protecting communities, and maintaining healthy ecosystems.

## Supporting Information Available

Prescribed burn detection method, burn area, burning season impact, maximum concentration, yearly premature death, and seasonal data (PDF)

## References

[ref1] Burke M.; Driscoll A.; Heft-Neal S.; Xue J.; Burney J.; Wara M. The Changing Risk and Burden of Wildfire in the United States. Proc. Natl. Acad. Sci. U. S. A. 2021, 118 (2), e2011048118 10.1073/pnas.2011048118. 33431571 PMC7812759

[ref2] Kolden C. We’re Not Doing Enough Prescribed Fire in the Western United States to Mitigate Wildfire Risk. Fire 2019, 2 (2), 30 10.3390/fire2020030.

[ref3] Keywood M.; Kanakidou M.; Stohl A.; Dentener F.; Grassi G.; Meyer C. P.; Torseth K.; Edwards D.; Thompson A. M.; Lohmann U.; Burrows J. Fire in the Air: Biomass Burning Impacts in a Changing Climate. Crit Rev. Environ. Sci. Technol. 2013, 43 (1), 40–83. 10.1080/10643389.2011.604248.

[ref4] Collins L.; Trouvé R.; Baker P. J.; Cirulus B.; Nitschke C. R.; Nolan R. H.; Smith L.; Penman T. D. Fuel Reduction Burning Reduces Wildfire Severity during Extreme Fire Events in South-Eastern Australia. J. Environ. Manage 2023, 343, 118171 10.1016/j.jenvman.2023.118171. 37245307

[ref5] Schoennagel T.; Balch J. K.; Brenkert-Smith H.; Dennison P. E.; Harvey B. J.; Krawchuk M. A.; Mietkiewicz N.; Morgan P.; Moritz M. A.; Rasker R.; Turner M. G.; Whitlock C. Adapt to More Wildfire in Western North American Forests as Climate Changes. Proc. Natl. Acad. Sci. U. S. A. 2017, 114 (18), 4582–4590. 10.1073/pnas.1617464114. 28416662 PMC5422781

[ref6] Jaffe D. A.; O’Neill S. M.; Larkin N. K.; Holder A. L.; Peterson D. L.; Halofsky J. E.; Rappold A. G. Wildfire and Prescribed Burning Impacts on Air Quality in the United States. J. Air Waste Manage Assoc 2020, 70 (6), 583–615. 10.1080/10962247.2020.1749731. PMC7932990 32240055

[ref7] Wu C.-M.; Song C. C.; Chartier R.; Kremer J.; Naeher L.; Adetona O. Characterization of Occupational Smoke Exposure among Wildland Firefighters in the Midwestern United States. Environ. Res. 2021, 193, 110541 10.1016/j.envres.2020.110541. 33249041

[ref8] Afrin S.; Garcia-Menendez F. The Influence of Prescribed Fire on Fine Particulate Matter Pollution in the Southeastern United States. Geophys. Res. Lett. 2020, 47 (15), e2020GL088988 10.1029/2020GL088988.

[ref9] Williamson G. J.; Bowman D. M. J. S.; Price O. F.; Henderson S. B.; Johnston F. H. A Transdisciplinary Approach to Understanding the Health Effects of Wildfire and Prescribed Fire Smoke Regimes. Environmental Research Letters 2016, 11 (12), 125009 10.1088/1748-9326/11/12/125009.

[ref10] Ravi V.; Vaughan J. K.; Wolcott M. P.; Lamb B. K. Impacts of Prescribed Fires and Benefits from Their Reduction for Air Quality, Health, and Visibility in the Pacific Northwest of the United States. J. Air Waste Manage Assoc 2019, 69 (3), 289–304. 10.1080/10962247.2018.1526721. 30252621

[ref11] Huff A. K.; Kondragunta S.; Zhang H.; Laszlo I.; Zhou M.; Caicedo V.; Delgado R.; Levy R. Tracking Smoke from a Prescribed Fire and Its Impacts on Local Air Quality Using Temporally Resolved GOES-16 ABI Aerosol Optical Depth (AOD). J. Atmos Ocean Technol. 2021, 38 (5), 963–976. 10.1175/JTECH-D-20-0162.1.

[ref12] U.S. EPA. Comparative Assessment of the Impacts of Prescribed Fire Versus Wildfire (CAIF): A Case Study in the Western U.S.; EPA: Washington, DC, 2021.

[ref13] Chen G.; Guo Y.; Yue X.; Tong S.; Gasparrini A.; Bell M. L.; Armstrong B.; Schwartz J.; Jaakkola J. J. K.; Zanobetti A.; Lavigne E.; Nascimento Saldiva P. H.; Kan H.; Royé D.; Milojevic A.; Overcenco A.; Urban A.; Schneider A.; Entezari A.; Vicedo-Cabrera A. M.; Zeka A.; Tobias A.; Nunes B.; Alahmad B.; Forsberg B.; Pan S.-C.; Íñiguez C.; Ameling C.; De la Cruz Valencia C.; Åström C.; Houthuijs D.; Van Dung D.; Samoli E.; Mayvaneh F.; Sera F.; Carrasco-Escobar G.; Lei Y.; Orru H.; Kim H.; Holobaca I.-H.; Kyselý J.; Teixeira J. P.; Madureira J.; Katsouyanni K.; Hurtado-Díaz M.; Maasikmets M.; Ragettli M. S.; Hashizume M.; Stafoggia M.; Pascal M.; Scortichini M.; de Sousa Zanotti Stagliorio Coêlho M.; Valdés Ortega N.; Ryti N. R. I.; Scovronick N.; Matus P.; Goodman P.; Garland R. M.; Abrutzky R.; Garcia S. O.; Rao S.; Fratianni S.; Dang T. N.; Colistro V.; Huber V.; Lee W.; Seposo X.; Honda Y.; Guo Y. L.; Ye T.; Yu W.; Abramson M. J.; Samet J. M.; Li S. Mortality Risk Attributable to Wildfire-Related PM2·5 Pollution: A Global Time Series Study in 749 Locations. Lancet Planet Health 2021, 5 (9), e579–e587. 10.1016/S2542-5196(21)00200-X. 34508679

[ref14] O’Dell K.; Bilsback K.; Ford B.; Martenies S. E.; Magzamen S.; Fischer E. V.; Pierce J. R. Estimated Mortality and Morbidity Attributable to Smoke Plumes in the United States: Not Just a Western US Problem. Geohealth 2021, 5 (9), e2021GH000457 10.1029/2021GH000457. PMC8420710 34504989

[ref15] Afrin S.; Garcia-Menendez F. Potential Impacts of Prescribed Fire Smoke on Public Health and Socially Vulnerable Populations in a Southeastern U.S. State. Science of The Total Environment 2021, 794, 148712 10.1016/j.scitotenv.2021.148712. 34323750

[ref16] Carter T. S.; Heald C. L.; Selin N. E. Large Mitigation Potential of Smoke PM _2.5_ in the US from Human-Ignited Fires. Environmental Research Letters 2023, 18 (1), 014002 10.1088/1748-9326/aca91f.

[ref17] Johnson Gaither C.; Afrin S.; Garcia-Menendez F.; Odman M. T.; Huang R.; Goodrick S.; Ricardo da Silva A. African American Exposure to Prescribed Fire Smoke in Georgia, USA. Int. J. Environ. Res. Public Health 2019, 16 (17), 3079 10.3390/ijerph16173079. 31450603 PMC6747108

[ref18] Li Z.; Maji K. J.; Hu Y.; Vaidyanathan A.; O’Neill S. M.; Odman M. T.; Russell A. G. An Analysis of Prescribed Fire Activities and Emissions in the Southeastern United States from 2013 to 2020. Remote Sens (Basel) 2023, 15 (11), 2725 10.3390/rs15112725. PMC11296730 39100105

[ref19] Huang R.; Zhang X.; Chan D.; Kondragunta S.; Russell A. G.; Odman M. T. Burned Area Comparisons Between Prescribed Burning Permits in Southeastern United States and Two Satellite-Derived Products. Journal of Geophysical Research: Atmospheres 2018, 123 (9), 4746–4757. 10.1029/2017JD028217.

[ref20] Eyth A. National Emission Inventory (NEI) 2016 Modeling Platform Version 2; CMAS Data Warehouse, 2021.

[ref21] Wilkins J. L.; Pouliot G.; Foley K.; Appel W.; Pierce T. The Impact of US Wildland Fires on Ozone and Particulate Matter: A Comparison of Measurements and CMAQ Model Predictions from 2008 to 2012. Int. J. Wildland Fire 2018, 27 (10), 684 10.1071/WF18053. PMC7788068 33424209

[ref22] Koplitz S. N.; Nolte C. G.; Sabo R. D.; Clark C. M.; Horn K. J.; Thomas R. Q.; Newcomer-Johnson T. A. The Contribution of Wildland Fire Emissions to Deposition in the U S: Implications for Tree Growth and Survival in the Northwest. Environmental Research Letters 2021, 16 (2), 024028 10.1088/1748-9326/abd26e. PMC7970516 33747119

[ref23] Herron-Thorpe F. L.; Mount G. H.; Emmons L. K.; Lamb B. K.; Jaffe D. A.; Wigder N. L.; Chung S. H.; Zhang R.; Woelfle M. D.; Vaughan J. K. Air Quality Simulations of Wildfires in the Pacific Northwest Evaluated with Surface and Satellite Observations during the Summers of 2007 and 2008. Atmos Chem. Phys. 2014, 14 (22), 12533–12551. 10.5194/acp-14-12533-2014.

[ref24] Michael R.; Mirabelli M. C.; Vaidyanathan A. Public Health Applications of Historical Smoke Forecasts: An Evaluation of Archived BlueSky Data for the Coterminous United States, 2015–2018. Comput. Geosci 2023, 171, 105267 10.1016/j.cageo.2022.105267. PMC11296727 39100411

[ref25] Strand T. M.; Larkin N.; Craig K. J.; Raffuse S.; Sullivan D.; Solomon R.; Rorig M.; Wheeler N.; Pryden D. Analyses of BlueSky Gateway PM _2.5_ Predictions during the 2007 Southern and 2008 Northern California Fires. J. Geophys. Res.: Atmos. 2012, 117 (D17), 017627 10.1029/2012JD017627.

[ref26] Ottmar R. D.; Sandberg D. V.; Riccardi C. L.; Prichard S. J. An Overview of the Fuel Characteristic Classification System — Quantifying, Classifying, and Creating Fuelbeds for Resource PlanningThis Article Is One of a Selection of Papers Published in the Special Forum on the Fuel Characteristic Classification System. Canadian Journal of Forest Research 2007, 37 (12), 2383–2393. 10.1139/X07-077.

[ref27] Prichard S. J.; O’Neill S. M.; Eagle P.; Andreu A. G.; Drye B.; Dubowy J.; Urbanski S.; Strand T. M. Wildland Fire Emission Factors in North America: Synthesis of Existing Data, Measurement Needs and Management Applications. Int. J. Wildland Fire 2020, 29 (2), 132 10.1071/WF19066.

[ref28] Otte T. L.; Pleim J. E. The Meteorology-Chemistry Interface Processor (MCIP) for the CMAQ Modeling System: Updates through MCIPv3.4.1. Geosci Model Dev 2010, 3 (1), 243–256. 10.5194/gmd-3-243-2010.

[ref29] Gantt B.; Kelly J. T.; Bash J. O. Updating Sea Spray Aerosol Emissions in the Community Multiscale Air Quality (CMAQ) Model Version 5.0.2. Geosci Model Dev 2015, 8 (11), 3733–3746. 10.5194/gmd-8-3733-2015.

[ref30] Kang D.; Pickering K. E.; Allen D. J.; Foley K. M.; Wong D. C.; Mathur R.; Roselle S. J. Simulating Lightning NO Production in CMAQv5.2: Evolution of Scientific Updates. Geosci Model Dev 2019, 12 (7), 3071–3083. 10.5194/gmd-12-3071-2019. 32206207 PMC7087390

[ref31] EPA. National Emissions Inventory (NEI) Data; EPA, 2011.

[ref32] Bash J. O.; Baker K. R.; Beaver M. R. Evaluation of Improved Land Use and Canopy Representation in BEIS v3.61 with Biogenic VOC Measurements in California. Geosci Model Dev 2016, 9 (6), 2191–2207. 10.5194/gmd-9-2191-2016.

[ref33] USEPA. 2011 Version 6 Air Emissions Modeling Platforms. https://www.epa.gov/air-emissions-modeling/2011-version-6-air-emissions-modeling-platforms (accessed 2024-05-21).

[ref34] Friberg M. D.; Zhai X.; Holmes H. A.; Chang H. H.; Strickland M. J.; Sarnat S. E.; Tolbert P. E.; Russell A. G.; Mulholland J. A. Method for Fusing Observational Data and Chemical Transport Model Simulations To Estimate Spatiotemporally Resolved Ambient Air Pollution. Environ. Sci. Technol. 2016, 50 (7), 3695–3705. 10.1021/acs.est.5b05134. 26923334

[ref35] Senthilkumar N.; Gilfether M.; Metcalf F.; Russell A. G.; Mulholland J. A.; Chang H. H. Application of a Fusion Method for Gas and Particle Air Pollutants between Observational Data and Chemical Transport Model Simulations Over the Contiguous United States for 2005–2014. Int. J. Environ. Res. Public Health 2019, 16 (18), 3314 10.3390/ijerph16183314. 31505818 PMC6765984

[ref36] U.S. EPA. Air Data Home-Pre-Generated Data Files. https://aqs.epa.gov/aqsweb/airdata/download_files.html.

[ref37] Neumann J. E.; Amend M.; Anenberg S.; Kinney P. L.; Sarofim M.; Martinich J.; Lukens J.; Xu J.-W.; Roman H. Estimating PM2.5-Related Premature Mortality and Morbidity Associated with Future Wildfire Emissions in the Western US. Environmental Research Letters 2021, 16 (3), 035019 10.1088/1748-9326/abe82b. PMC8048092 33868453

[ref38] Clarke H.; Cirulis B.; Borchers-Arriagada N.; Bradstock R.; Price O.; Penman T. Health Costs of Wildfire Smoke to Rise under Climate Change. NPJ. Clim Atmos Sci. 2023, 6 (1), 102 10.1038/s41612-023-00432-0.

[ref39] Lou S.; Liu Y.; Bai Y.; Li F.; Lin G.; Xu L.; Liu Z.; Chen Y.; Dong X.; Zhao M.; Wang L.; Jin M.; Wang C.; Cai W.; Gong P.; Luo Y. Projections of Mortality Risk Attributable to Short-Term Exposure to Landscape Fire Smoke in China, 2021–2100: A Health Impact Assessment Study. Lancet Planet Health 2023, 7 (10), e841–e849. 10.1016/S2542-5196(23)00192-4. 37821162 PMC10620468

[ref40] Maji K. J. Substantial Changes in PM2.5 Pollution and Corresponding Premature Deaths across China during 2015–2019: A Model Prospective. Science of The Total Environment 2020, 729, 138838 10.1016/j.scitotenv.2020.138838. 32361442

[ref41] Salonen H.; Salthammer T.; Morawska L. Human Exposure to Ozone in School and Office Indoor Environments. Environ. Int. 2018, 119, 503–514. 10.1016/j.envint.2018.07.012. 30053738

[ref42] Gentner D. R.; Jathar S. H.; Gordon T. D.; Bahreini R.; Day D. A.; El Haddad I.; Hayes P. L.; Pieber S. M.; Platt S. M.; de Gouw J.; Goldstein A. H.; Harley R. A.; Jimenez J. L.; Prévôt A. S. H.; Robinson A. L. Review of Urban Secondary Organic Aerosol Formation from Gasoline and Diesel Motor Vehicle Emissions. Environ. Sci. Technol. 2017, 51 (3), 1074–1093. 10.1021/acs.est.6b04509. 28000440

[ref43] Wang Y.; Xiao S.; Zhang Y.; Chang H.; Martin R. V.; Van Donkelaar A.; Gaskins A.; Liu Y.; Liu P.; Shi L. Long-Term Exposure to PM2.5 Major Components and Mortality in the Southeastern United States. Environ. Int. 2022, 158, 106969 10.1016/j.envint.2021.106969. 34741960 PMC9190768

[ref44] Pan S.; Gan L.; Jung J.; Yu W.; Roy A.; Diao L.; Jeon W.; Souri A. H.; Gao H. O.; Choi Y. Quantifying the Premature Mortality and Economic Loss from Wildfire-Induced PM2.5 in the Contiguous U.S. Science of The Total Environment 2023, 875, 162614 10.1016/j.scitotenv.2023.162614. 36871727

[ref45] Aguilera R.; Corringham T.; Gershunov A.; Benmarhnia T. Wildfire Smoke Impacts Respiratory Health More than Fine Particles from Other Sources: Observational Evidence from Southern California. Nat. Commun. 2021, 12 (1), 1493 10.1038/s41467-021-21708-0. 33674571 PMC7935892

[ref46] Bell M. L. Ozone and Short-Term Mortality in 95 US Urban Communities, 1987–2000. JAMA 2004, 292 (19), 2372 10.1001/jama.292.19.2372. 15547165 PMC3546819

[ref47] Bell M. L.; Dominici F.; Samet J. M. A Meta-Analysis of Time-Series Studies of Ozone and Mortality With Comparison to the National Morbidity, Mortality, and Air Pollution Study. Epidemiology 2005, 16 (4), 436–445. 10.1097/01.ede.0000165817.40152.85. 15951661 PMC3581312

[ref48] Institute for Health Metrics and Evaluation (IHME). Global Burden of Disease (GBD) data and tools guide: Appendix 1: Full list of locations, diseases, injuries, and risk factors.

[ref49] Emery C.; Liu Z.; Russell A. G.; Odman M. T.; Yarwood G.; Kumar N. Recommendations on Statistics and Benchmarks to Assess Photochemical Model Performance. J. Air Waste Manage Assoc 2017, 67 (5), 582–598. 10.1080/10962247.2016.1265027. 27960634

[ref50] Watson G. L.; Telesca D.; Reid C. E.; Pfister G. G.; Jerrett M. Machine Learning Models Accurately Predict Ozone Exposure during Wildfire Events. Environ. Pollut. 2019, 254, 112792 10.1016/j.envpol.2019.06.088. 31421571

[ref51] Wang W.; Liu X.; Bi J.; Liu Y. A Machine Learning Model to Estimate Ground-Level Ozone Concentrations in California Using TROPOMI Data and High-Resolution Meteorology. Environ. Int. 2022, 158, 106917 10.1016/j.envint.2021.106917. 34624589

[ref52] Di Q.; Amini H.; Shi L.; Kloog I.; Silvern R.; Kelly J.; Sabath M. B.; Choirat C.; Koutrakis P.; Lyapustin A.; Wang Y.; Mickley L. J.; Schwartz J. An Ensemble-Based Model of PM2.5 Concentration across the Contiguous United States with High Spatiotemporal Resolution. Environ. Int. 2019, 130, 104909 10.1016/j.envint.2019.104909. 31272018 PMC7063579

[ref53] Huang R.; Lal R.; Qin M.; Hu Y.; Russell A. G.; Odman M. T.; Afrin S.; Garcia-Menendez F.; O’Neill S. M. Application and Evaluation of a Low-Cost PM Sensor and Data Fusion with CMAQ Simulations to Quantify the Impacts of Prescribed Burning on Air Quality in Southwestern Georgia, USA. J. Air Waste Manage Assoc 2021, 71 (7), 815–829. 10.1080/10962247.2021.1924311. 33914671

[ref54] Population Reference Bureau (PRB). United State Indicators: Total Deaths. https://www.prb.org/usdata/indicator/deaths/table/?geos=GA.

[ref55] Wiedinmyer C.; Kimura Y.; McDonald-Buller E. C.; Emmons L. K.; Buchholz R. R.; Tang W.; Seto K.; Joseph M. B.; Barsanti K. C.; Carlton A. G.; Yokelson R. The Fire Inventory from NCAR Version 2.5: An Updated Global Fire Emissions Model for Climate and Chemistry Applications. Geosci Model Dev 2023, 16 (13), 3873–3891. 10.5194/gmd-16-3873-2023.

[ref56] Lu X.; Zhang X.; Li F.; Cochrane M. A. Investigating Smoke Aerosol Emission Coefficients Using MODIS Active Fire and Aerosol Products: A Case Study in the CONUS and Indonesia. J. Geophys Res. Biogeosci 2019, 124 (6), 1413–1429. 10.1029/2018JG004974.

[ref57] Nowell H. K.; Holmes C. D.; Robertson K.; Teske C.; Hiers J. K. A New Picture of Fire Extent, Variability, and Drought Interaction in Prescribed Fire Landscapes: Insights From Florida Government Records. Geophys. Res. Lett. 2018, 45 (15), 7874–7884. 10.1029/2018GL078679. 31031448 PMC6474124

[ref58] Mangeon S.; Field R.; Fromm M.; McHugh C.; Voulgarakis A. Satellite versus Ground-Based Estimates of Burned Area: A Comparison between MODIS Based Burned Area and Fire Agency Reports over North America in 2007. Anthropocene Review 2016, 3 (2), 76–92. 10.1177/2053019615588790.

[ref59] Chuvieco E.; Mouillot F.; van der Werf G. R.; San Miguel J.; Tanase M.; Koutsias N.; García M.; Yebra M.; Padilla M.; Gitas I.; Heil A.; Hawbaker T. J.; Giglio L. Historical Background and Current Developments for Mapping Burned Area from Satellite Earth Observation. Remote Sens Environ 2019, 225, 45–64. 10.1016/j.rse.2019.02.013.

[ref60] Canadell J. G.; Meyer C. P.; Cook G. D.; Dowdy A.; Briggs P. R.; Knauer J.; Pepler A.; Haverd V. Multi-Decadal Increase of Forest Burned Area in Australia Is Linked to Climate Change. Nat. Commun. 2021, 12 (1), 6921 10.1038/s41467-021-27225-4. 34836974 PMC8626427

[ref61] EPD. Open Burning Rules for Georgia. https://epd.georgia.gov/air-protection-branch/open-burning-rules-georgia.

[ref62] Baker K. R.; Woody M. C.; Tonnesen G. S.; Hutzell W.; Pye H. O. T.; Beaver M. R.; Pouliot G.; Pierce T. Contribution of Regional-Scale Fire Events to Ozone and PM2.5 Air Quality Estimated by Photochemical Modeling Approaches. Atmos. Environ. 2016, 140, 539–554. 10.1016/j.atmosenv.2016.06.032.

[ref63] Ninneman M.; Jaffe D. A. The Impact of Wildfire Smoke on Ozone Production in an Urban Area: Insights from Field Observations and Photochemical Box Modeling. Atmos. Environ. 2021, 267, 118764 10.1016/j.atmosenv.2021.118764.

[ref64] Qian Y.; Henneman L. R. F.; Mulholland J. A.; Russell A. G. Empirical Development of Ozone Isopleths: Applications to Los Angeles. Environ. Sci. Technol. Lett. 2019, 6 (5), 294–299. 10.1021/acs.estlett.9b00160.

[ref65] Liu Z.; Liu Y.; Murphy J. P.; Maghirang R. Contributions of Kansas Rangeland Burning to Ambient O3: Analysis of Data from 2001 to 2016. Science of The Total Environment 2018, 618, 1024–1031. 10.1016/j.scitotenv.2017.09.075. 29074244

[ref66] Cleary P. A.; Fuhrman N.; Schulz L.; Schafer J.; Fillingham J.; Bootsma H.; McQueen J.; Tang Y.; Langel T.; McKeen S.; Williams E. J.; Brown S. S. Ozone Distributions over Southern Lake Michigan: Comparisons between Ferry-Based Observations, Shoreline-Based DOAS Observations and Model Forecasts. Atmos Chem. Phys. 2015, 15 (9), 5109–5122. 10.5194/acp-15-5109-2015.

[ref67] Burley J. D.; Theiss S.; Bytnerowicz A.; Gertler A.; Schilling S.; Zielinska B. Surface Ozone in the Lake Tahoe Basin. Atmos. Environ. 2015, 109, 351–369. 10.1016/j.atmosenv.2015.02.001.

[ref68] Gantt B.; Sarwar G.; Xing J.; Simon H.; Schwede D.; Hutzell W. T.; Mathur R.; Saiz-Lopez A. The Impact of Iodide-Mediated Ozone Deposition and Halogen Chemistry on Surface Ozone Concentrations Across the Continental United States. Environ. Sci. Technol. 2017, 51 (3), 1458–1466. 10.1021/acs.est.6b03556. 28051851 PMC6145082

[ref69] Holm S. M.; Miller M. D.; Balmes J. R. Health Effects of Wildfire Smoke in Children and Public Health Tools: A Narrative Review. J. Expo Sci. Environ. Epidemiol 2021, 31 (1), 1–20. 10.1038/s41370-020-00267-4. 32952154 PMC7502220

[ref70] Reid C. E.; Brauer M.; Johnston F. H.; Jerrett M.; Balmes J. R.; Elliott C. T. Critical Review of Health Impacts of Wildfire Smoke Exposure. Environ. Health Perspect 2016, 124 (9), 1334–1343. 10.1289/ehp.1409277. 27082891 PMC5010409

[ref71] Gao Y.; Huang W.; Yu P.; Xu R.; Gasevic D.; Yue X.; Coêlho M. d. S. Z. S.; Saldiva P. H. N.; Guo Y.; Li S. Wildfire-Related PM2.5 and Cardiovascular Mortality: A Difference-in-Differences Analysis in Brazil. Environ. Pollut. 2024, 347, 123810 10.1016/j.envpol.2024.123810. 38493867

[ref72] Liu J. C.; Pereira G.; Uhl S. A.; Bravo M. A.; Bell M. L. A Systematic Review of the Physical Health Impacts from Non-Occupational Exposure to Wildfire Smoke. Environ. Res. 2015, 136, 120–132. 10.1016/j.envres.2014.10.015. 25460628 PMC4262561

[ref73] Hadley M. B.; Henderson S. B.; Brauer M.; Vedanthan R. Protecting Cardiovascular Health From Wildfire Smoke. Circulation 2022, 146 (10), 788–801. 10.1161/CIRCULATIONAHA.121.058058. 36067276

[ref74] Pullabhotla H. K.; Zahid M.; Heft-Neal S.; Rathi V.; Burke M. Global Biomass Fires and Infant Mortality. Proc. Natl. Acad. Sci. U. S. A. 2023, 120 (23), e2218210120 10.1073/pnas.2218210120. 37253010 PMC10266003

[ref75] Ma Y.; Zang E.; Liu Y.; Lu Y.; Krumholz H. M.; Bell M. L.; Chen K. Wildfire Smoke PM2.5 and Mortality in the Contiguous United States. medRxiv 2023, 10.1101/2023.01.31.23285059. PMC11459178 39316057

[ref76] O’Dell K.; Ford B.; Burkhardt J.; Magzamen S.; Anenberg S. C.; Bayham J.; Fischer E. V.; Pierce J. R. Outside in: The Relationship between Indoor and Outdoor Particulate Air Quality during Wildfire Smoke Events in Western US Cities. Environmental Research: Health 2023, 1 (1), 015003 10.1088/2752-5309/ac7d69.

[ref77] Childs M. L.; Li J.; Wen J.; Heft-Neal S.; Driscoll A.; Wang S.; Gould C. F.; Qiu M.; Burney J.; Burke M. Daily Local-Level Estimates of Ambient Wildfire Smoke PM _2.5_ for the Contiguous US. Environ. Sci. Technol. 2022, 56 (19), 13607–13621. 10.1021/acs.est.2c02934. 36134580

[ref78] Ye T.; Xu R.; Yue X.; Chen G.; Yu P.; Coêlho M. S. Z. S.; Saldiva P. H. N.; Abramson M. J.; Guo Y.; Li S. Short-Term Exposure to Wildfire-Related PM2.5 Increases Mortality Risks and Burdens in Brazil. Nat. Commun. 2022, 13 (1), 7651 10.1038/s41467-022-35326-x. 36496479 PMC9741581

[ref79] Laurent O.; Hu J.; Li L.; Kleeman M. J.; Bartell S. M.; Cockburn M.; Escobedo L.; Wu J. A Statewide Nested Case–Control Study of Preterm Birth and Air Pollution by Source and Composition: California, 2001–2008. Environ. Health Perspect 2016, 124 (9), 1479–1486. 10.1289/ehp.1510133. 26895492 PMC5010414

[ref80] Sánchez-García C.; Santín C.; Neris J.; Sigmund G.; Otero X. L.; Manley J.; González-Rodríguez G.; Belcher C. M.; Cerdà A.; Marcotte A. L.; Murphy S. F.; Rhoades C. C.; Sheridan G.; Strydom T.; Robichaud P. R.; Doerr S. H. Chemical Characteristics of Wildfire Ash across the Globe and Their Environmental and Socio-Economic Implications. Environ. Int. 2023, 178, 108065 10.1016/j.envint.2023.108065. 37562341

[ref81] Gkatzelis G. I.; Coggon M. M.; Stockwell C. E.; Hornbrook R. S.; Allen H.; Apel E. C.; Bela M. M.; Blake D. R.; Bourgeois I.; Brown S. S.; Campuzano-Jost P.; St. Clair J. M.; Crawford J. H.; Crounse J. D.; Day D. A.; DiGangi J. P.; Diskin G. S.; Fried A.; Gilman J. B.; Guo H.; Hair J. W.; Halliday H. S.; Hanisco T. F.; Hannun R.; Hills A.; Huey L. G.; Jimenez J. L.; Katich J. M.; Lamplugh A.; Lee Y. R.; Liao J.; Lindaas J.; McKeen S. A.; Mikoviny T.; Nault B. A.; Neuman J. A.; Nowak J. B.; Pagonis D.; Peischl J.; Perring A. E.; Piel F.; Rickly P. S.; Robinson M. A.; Rollins A. W.; Ryerson T. B.; Schueneman M. K.; Schwantes R. H.; Schwarz J. P.; Sekimoto K.; Selimovic V.; Shingler T.; Tanner D. J.; Tomsche L.; Vasquez K. T.; Veres P. R.; Washenfelder R.; Weibring P.; Wennberg P. O.; Wisthaler A.; Wolfe G. M.; Womack C. C.; Xu L.; Ball K.; Yokelson R. J.; Warneke C. Parameterizations of US Wildfire and Prescribed Fire Emission Ratios and Emission Factors Based on FIREX-AQ Aircraft Measurements. Atmos. Chem. Phys. 2024, 24 (2), 929–956. 10.5194/acp-24-929-2024.

[ref82] EPA. Comparative Assessment of the Impacts of Prescribed Fire Versus Wildfire (CAIF): A Case Study in the Western U.S.; EPA: Research Triangle Park, NC, 2021.

[ref83] Zanobetti A.; Schwartz J. The Effect of Fine and Coarse Particulate Air Pollution on Mortality: A National Analysis. Environ. Health Perspect 2009, 117 (6), 898–903. 10.1289/ehp.0800108. 19590680 PMC2702403

[ref84] Grant E.; Runkle J. D. Long-Term Health Effects of Wildfire Exposure: A Scoping Review. Journal of Climate Change and Health 2022, 6, 100110 10.1016/j.joclim.2021.100110.

[ref85] Paugam R.; Wooster M.; Freitas S.; Val Martin M. A Review of Approaches to Estimate Wildfire Plume Injection Height within Large-Scale Atmospheric Chemical Transport Models. Atmos Chem. Phys. 2016, 16 (2), 907–925. 10.5194/acp-16-907-2016.

[ref86] Briggs G. A. Plume Rise Predictions. In Lectures on Air Pollution and Environmental Impact Analyses; American Meteorological Society; American Meteorological Society: Boston, MA, 1982; pp. 59–111.

[ref87] Moisseeva N.; Stull R. Wildfire Smoke-Plume Rise: A Simple Energy Balance Parameterization. Atmos Chem. Phys. 2021, 21 (3), 1407–1425. 10.5194/acp-21-1407-2021.

[ref88] Pavlovic R.; Chen J.; Anderson K.; Moran M. D.; Beaulieu P.-A.; Davignon D.; Cousineau S. The FireWork Air Quality Forecast System with Near-Real-Time Biomass Burning Emissions: Recent Developments and Evaluation of Performance for the 2015 North American Wildfire Season. J. Air Waste Manage Assoc 2016, 66 (9), 819–841. 10.1080/10962247.2016.1158214. PMC5062048 26934496

[ref89] Garcia-Menendez F.; Hu Y.; Odman M. T. Simulating Smoke Transport from Wildland Fires with a Regional-Scale Air Quality Model: Sensitivity to Spatiotemporal Allocation of Fire Emissions. Science of The Total Environment 2014, 493, 544–553. 10.1016/j.scitotenv.2014.05.108. 24973934

[ref90] Cleland S. E.; West J. J.; Jia Y.; Reid S.; Raffuse S.; O’Neill S.; Serre M. L. Estimating Wildfire Smoke Concentrations during the October 2017 California Fires through BME Space/Time Data Fusion of Observed, Modeled, and Satellite-Derived PM _2.5_. Environ. Sci. Technol. 2020, 54 (21), 13439–13447. 10.1021/acs.est.0c03761. 33064454 PMC7894965

[ref91] Huang R.; Hu Y.; Russell A. G.; Mulholland J. A.; Odman M. T. The Impacts of Prescribed Fire on PM2.5 Air Quality and Human Health: Application to Asthma-Related Emergency Room Visits in Georgia, USA. Int. J. Environ. Res. Public Health 2019, 16 (13), 2312 10.3390/ijerph16132312. 31261860 PMC6651061

[ref92] Westerling A. L. Increasing Western US Forest Wildfire Activity: Sensitivity to Changes in the Timing of Spring. Philosophical Transactions of the Royal Society B: Biological Sciences 2016, 371 (1696), 20150178 10.1098/rstb.2015.0178. PMC4874415 27216510

[ref93] Bar-Massada A.; Stewart S. I.; Hammer R. B.; Mockrin M. H.; Radeloff V. C. Using Structure Locations as a Basis for Mapping the Wildland Urban Interface. J. Environ. Manage 2013, 128, 540–547. 10.1016/j.jenvman.2013.06.021. 23831676

[ref94] Hiers J. K.; O’Brien J. J.; Varner J. M.; Butler B. W.; Dickinson M.; Furman J.; Gallagher M.; Godwin D.; Goodrick S. L.; Hood S. M.; Hudak A.; Kobziar L. N.; Linn R.; Loudermilk E. L.; McCaffrey S.; Robertson K.; Rowell E. M.; Skowronski N.; Watts A. C.; Yedinak K. M. Prescribed Fire Science: The Case for a Refined Research Agenda. Fire Ecology 2020, 16 (1), 11 10.1186/s42408-020-0070-8.

[ref95] Long J.; Drury S.; Evans S.; Maxwell C.; Scheller R. Comparing Smoke Emissions and Impacts under Alternative Forest Management Regimes. Ecology and Society 2022, 27 (4), art26 10.5751/ES-13553-270426.

[ref96] Eliott M. G.; Venn T. J.; Lewis T.; Farrar M.; Srivastava S. K. A Prescribed Fire Cost Model for Public Lands in South-East Queensland. For Policy Econ 2021, 132, 102579 10.1016/j.forpol.2021.102579.

[ref97] Price O. F.; Pausas J. G.; Govender N.; Flannigan M.; Fernandes P. M.; Brooks M. L.; Bird R. B. Global Patterns in Fire Leverage: The Response of Annual Area Burnt to Previous Fire. Int. J. Wildland Fire 2015, 24 (3), 297 10.1071/WF14034.

[ref98] Sacks J. D.; Holder A. L.; Rappold A. G.; Vaidyanathan A. At the Intersection: Protecting Public Health from Smoke While Addressing the U.S. Wildfire Crisis. Am. J. Respir Crit Care Med. 2023, 208 (7), 755–757. 10.1164/rccm.202304-0744VP. 37579300 PMC10563182

