# Supplementary material for: Estimated Impacts of Prescribed Fires on Air Quality and Premature Deaths in Georgia and Surrounding Areas in the US, 2015–2020
Source: Environ Sci Technol. 2024 Jun 29;58(28):12343–55. doi: 10.1021/acs.est.4c00890 (PMC11256750; doi:10.1021/acs.est.4c00890)
Supplement: Supplementary file 1 — es4c00890_si_001.pdf [file es4c00890_si_001.pdf]

Estimated Impacts of Prescribed Fires on Air Quality and Premature Deaths in Georgia and Surrounding Areas in the US, 2015-2020

Kamal J. Maji[1], Zongrun Li[1], Ambarish Vaidyanathan[1,2], Yongtao Hu[1], Jennifer D. Stowell[3], Chad Milando[3], Gregory Wellenius[3], Patrick L. Kinney[3], Armistead G. Russell[1], and M. Talat Odman[1,*]

[1]*School of Civil and Environmental Engineering, Georgia Institute of Technology, Atlanta, Georgia, USA*
[2]*National Center for Environmental Health, Centers for Disease Control and Prevention, Atlanta, Georgia, USA*
[3]*School of Public Health, Boston University, Boston, Massachusetts, USA*

Number of pages: 19
Number of figures: 12
Number of tables: 12

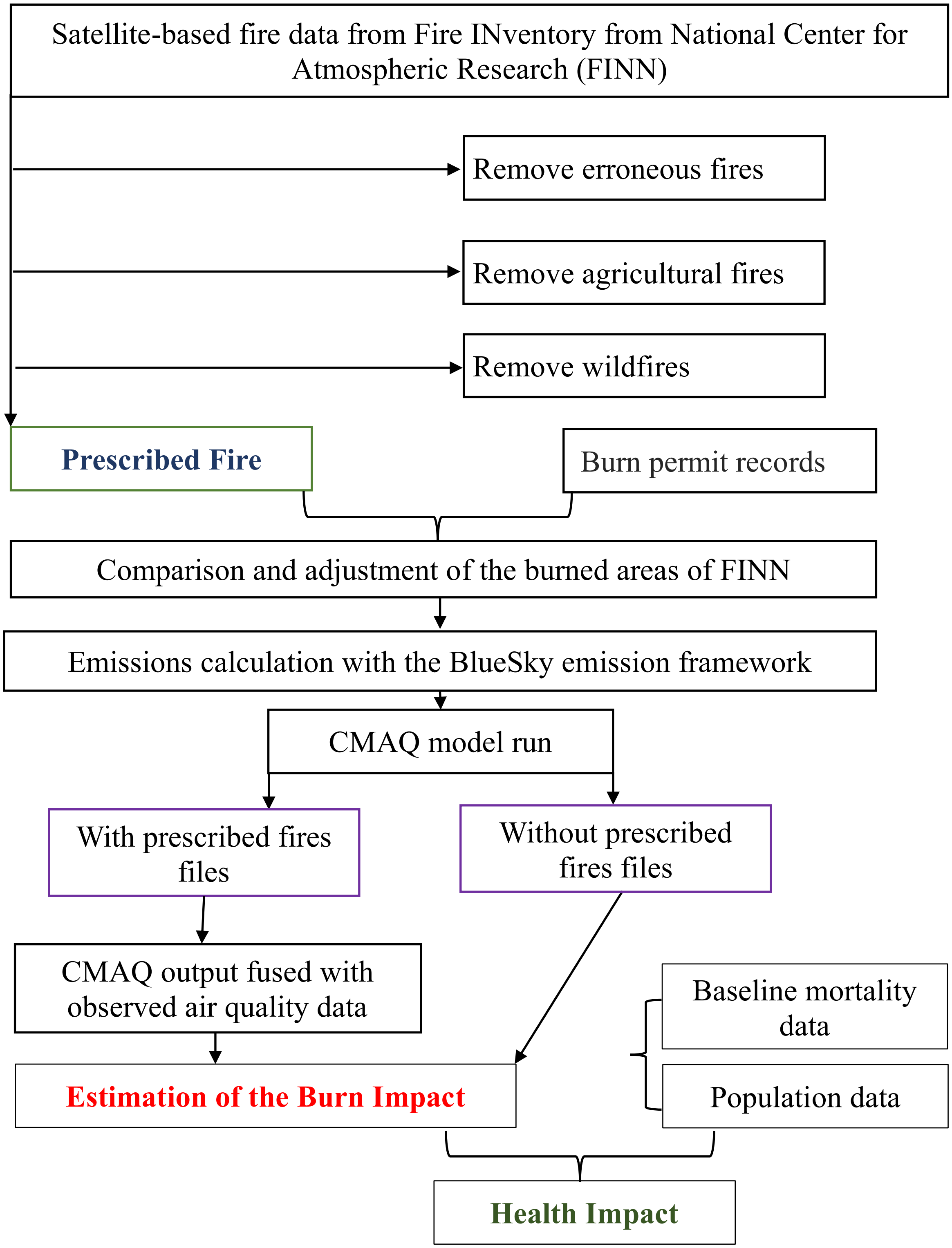

Satellite-based fire data from Fire INventory from National Center for Atmospheric Research (FINN)
Remove erroneous fires
Remove agricultural fires
Remove wildfires
Prescribed Fire
Burn permit records
Comparison and adjustment of the burned areas of FINN
Emissions calculation with the BlueSky emission framework
CMAQ model run
With prescribed fires files
Without prescribed fires files
CMAQ output fused with observed air quality data
Baseline mortality data
Estimation of the Burn Impact
Population data
Health Impact

Fig. S1. A simplified flow chart of the prescribed burn-associated air quality and health impact assessment methodology

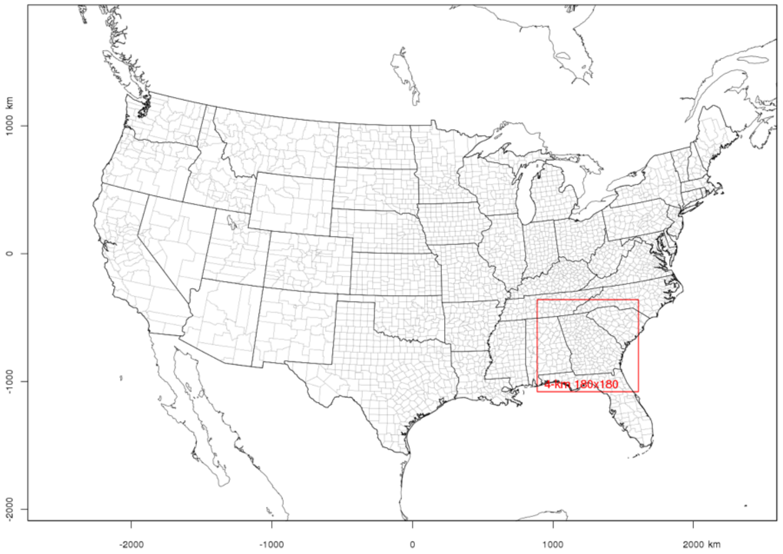

Figure S2: The red square box is the study domain.

Table S1. The relative risk (RR) (with 95% CI) used for premature death analysis.

Health endpoints	Pollutant	RR	β=ln(RR)/X<br>X=10 for $PM_{2.5}$ and 18.75 for MDA8-O_3	Source
All-cause mortality	Daily average $PM_{2.5}$	1·019 (1·016–1·022)	0.00188218 (0.00158733–0.00217615)	Chen et al (2021)
Cardiovascular mortality		1·017 (1·012–1·021)	0.00168571 (0.00119286–0.00207825)	
Respiratory mortality		1·019 (1·013–1·025)	0.00188218 (0.00129162–0.00246926)	
All-cause mortality	Daily average ozone	1.0087 (1.0055-1.0118)	0.00046199 (0.00029253–0.00062565)	Bell et al., (2005)
Cardiovascular mortality		1.0111 (1.0068-1.0153)	0.00058874 (0.00036144–0.00080982)	
Respiratory mortality		1.0064 (1.0031-1.0098)	0.00034025 (0.00016508–0.00052012)	Bell et al., (2004).

The daily ozone impact was modified for MDA8-O_3 impact based on the relationship between different ozone metrics [15:8 for the MDA8-O_3:daily average O_3] (Levy et al, 2001; Stieb et al,

2002). For example, a 10-ppb increase in the daily average ozone concentration corresponds to approximately 18.75-ppb increase in the MDA8-O_3.

S1.1. Data-Fusion:

Here, we breakdown the data-fusion approach (Friberg et al., 2016):

Ambient monitor observations provide very limited spatial information and information on temporal variation that decreases with increasing distance from monitors. CMAQ simulations, on the other hand, provide information that is independent of observations. The approach to fusing observations and CMAQ simulations involves three steps.

Interpolated Observation Method (FC_1):

To obtain pollutant estimates over space (*x*) and time (*t*) with temporal variation driven by monitor data, we first normalize daily observations (OBS_m) at each monitor (*m*) to annual mean levels ($\overline{OBS_m}$). Next, normalized data are spatially interpolated by the *krig* method described below. Lastly, the interpolated field is denormalized using the CMAQ annual field adjusted to the annual mean observations ($\overline{FC}$), also described below. Eq 1 describes this procedure for estimating daily concentration fields with temporal variation driven by observations and spatial structure governed by the adjusted annual mean CMAQ field.

$$FC_1(x,t) = \left(\frac{OBS_m(t)}{\overline{OBS_m}}\right)_{krig} \times \overline{FC(x)} \qquad (1)$$

Daily spatial interpolation of the normalized observations was performed by ordinary kriging. Normalization prior to kriging provides a smoother surface necessary with limited monitor coverage of the spatial domain. A more detailed spatial structure is obtained in denormalization using the mean CMAQ field.

Regression of annual mean measurements($\overline{OBS_m}$) and CMAQ simulations at monitor locations ($\overline{C_m}$) provides parameters for an annual mean pollutant field model that captures the spatial pattern of emissions and annual effects of meteorological variables, correcting for CMAQ annual biases (eq 2).

$$\overline{FC(x)} = \alpha_{year} \times \overline{C(x)}^{\beta} \quad (2)$$

Here, the overbar indicates temporal averaging (annual), β is a parameter derived for all years, and α_{year} is a regression parameter derived for each year. Spatial misalignment of measurements (point) and CMAQ simulations (4 × 4 km) and a changing network of monitors over time are factors that contribute to model instability and led to the use of more constrained models (e.g., nonvarying β) and zero intercepts. Linear models with slopes and intercepts varying each year provided similar results, although negative intercepts and greater variability across years were found.

Scaled CMAQ Method (FC_2):

Second, we develop fused concentration fields using adjusted daily CMAQ simulations, correcting for annual and seasonal biases (FC2) (eq 3).

$$FC_2(x,t) = C(s,t) \times \left(\frac{\overline{FC(x)}}{\overline{C(x)}}\right) \times \beta_{season}\,(t) \quad (3)$$

Here, β_{season} is the seasonal correction function. Analysis of the differential pattern between the observations and CMAQ simulations across species showed the seasonal component could be described and minimized using a sinusoidal cycle. The seasonal bias, which follows a sinusoidal variation, was modeled as a smooth trigonometric function (eq 4) with two fitted parameters: amplitude (A) and day of peak correction (t_{max}).

$$\beta_{season}(t) = e^{A\times\cos\left[\frac{2\pi}{365.25}(t-t_{max})\right]} \quad (4)$$

Use of this correction factor removed seasonal trends in the residual errors of the FC_2.

Optimized Fused Fields (FC_{opt}):

Third, we combine these to produce optimized fused concentration fields (C^{DF}) by computing a weighted average with the weight depending on the spatial autocorrelation of observations (which governs how well FC1 predicts temporal variance) and the correlation between observations and CMAQ simulations (which governs how well FC2 predicts temporal variance).

$$C^{DF}(x,t) = W(x,t) \times FC_1(x,t) + (1 - W(x,t)) \times FC_2(x,t) \quad (5)$$

W is an average A weighting factor, spatial fields for the study period. The optimized field resembles the FC_1 field near observations (where the weighting is large) and the FC_2 field far from observations (where the weighting is small).

Finally, we combine eq(1) to eq(4) put it in the eq(5) and represent in the manuscript.

$$C_{all}^{DF}(x,t) = \left[W(x,t) \times \left(\frac{OBS_m(t)}{\overline{OBS_m}} \right)_{krig} \times \overline{FC(x)} \right]$$

$$+ \left[\left(1 - W(x,t)\right) \times C_{all}^{s}(x,t) \times \left(\frac{\overline{FC(x)}}{\overline{C_{all}^{s}(x)}} \right) \times \beta_{season}(t) \right]$$

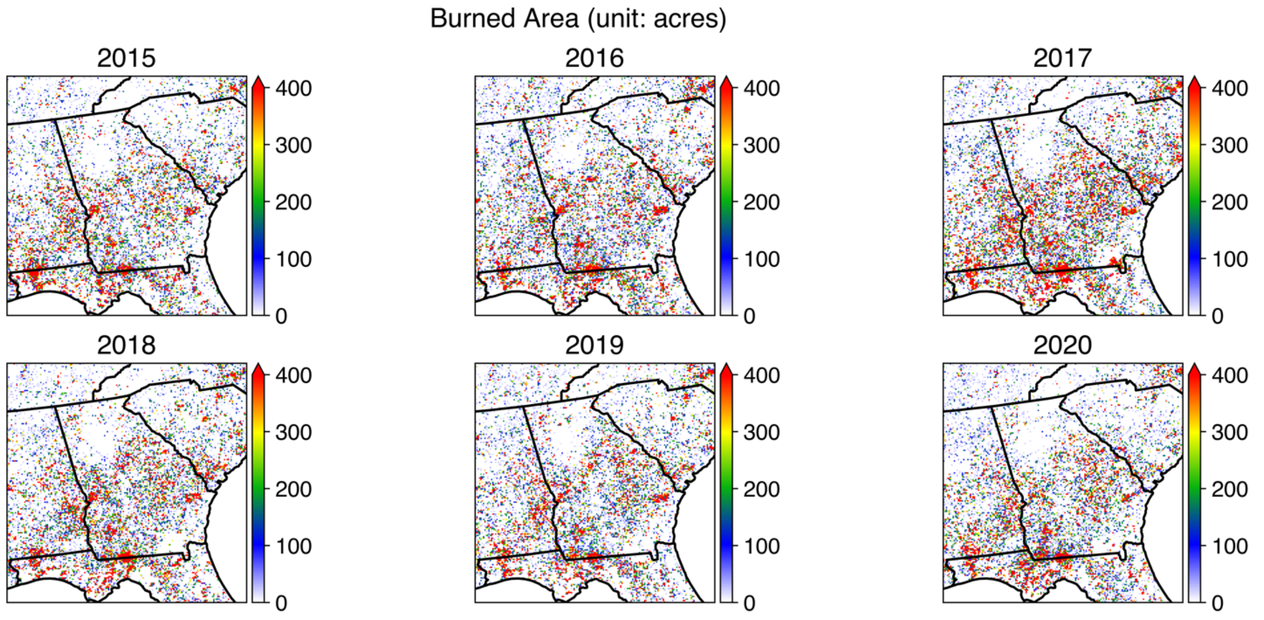

Fig S3. Spatial distribution of yearly prescribed burned area by adjusted FINN (unit: acres).

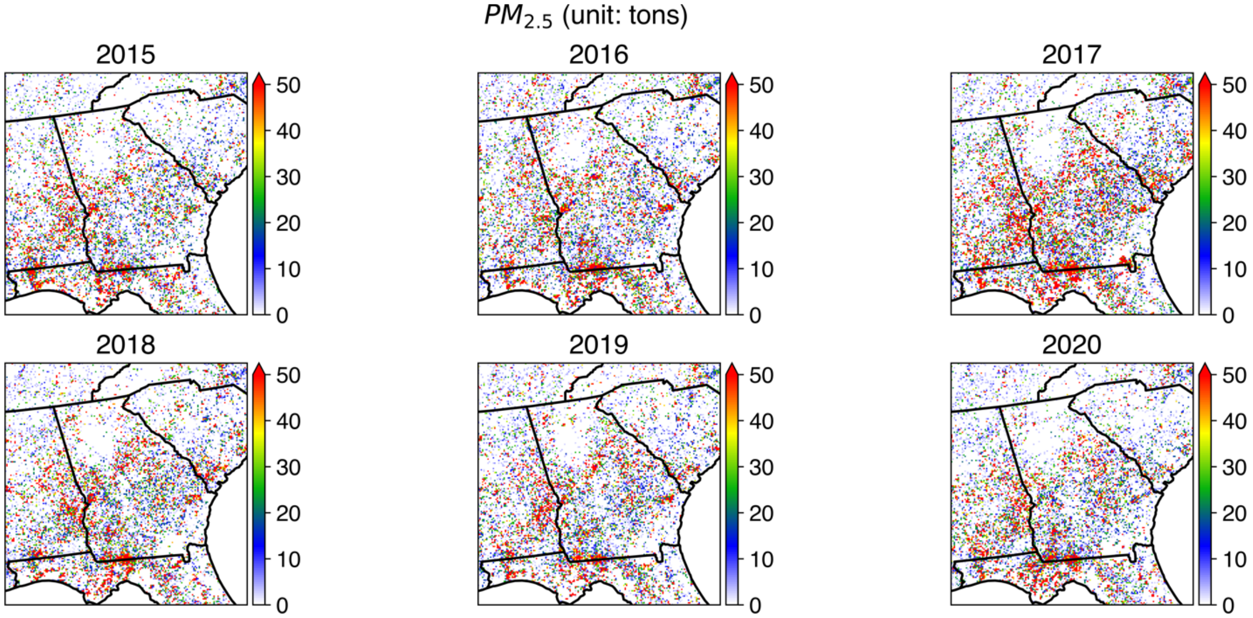

Fig S4. Spatial distribution of yearly prescribed burn emitted $PM_{2.5}$ (unit: tons).

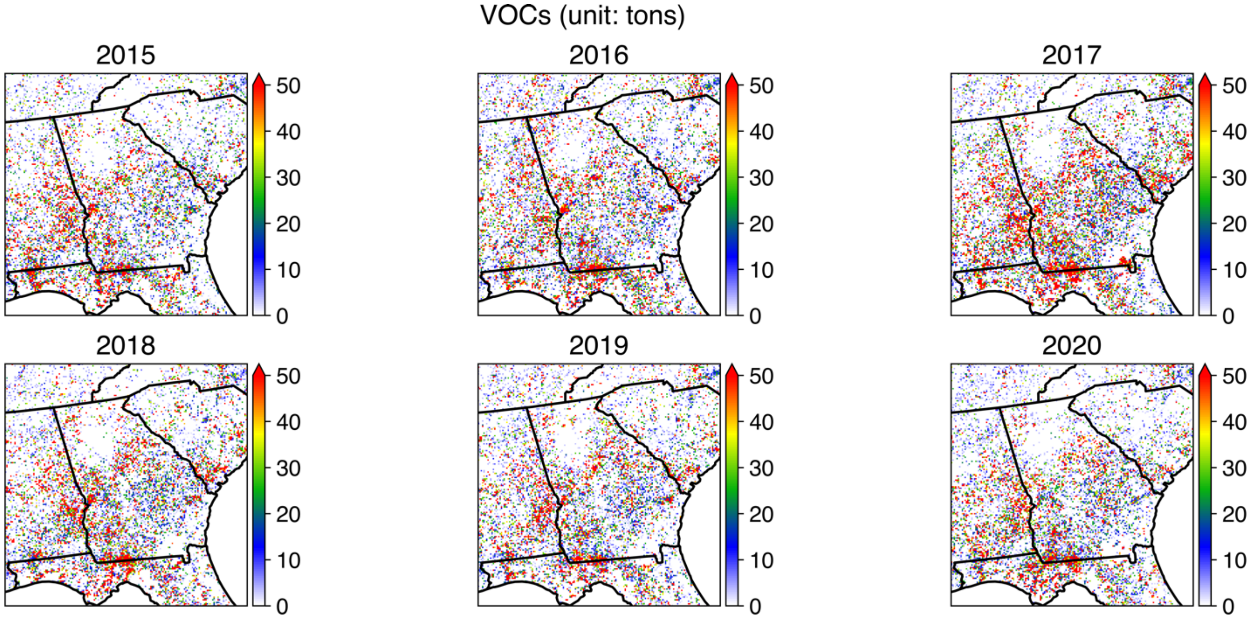

Fig S5. Spatial distribution of yearly prescribed burn emitted VOCs (unit: tons).

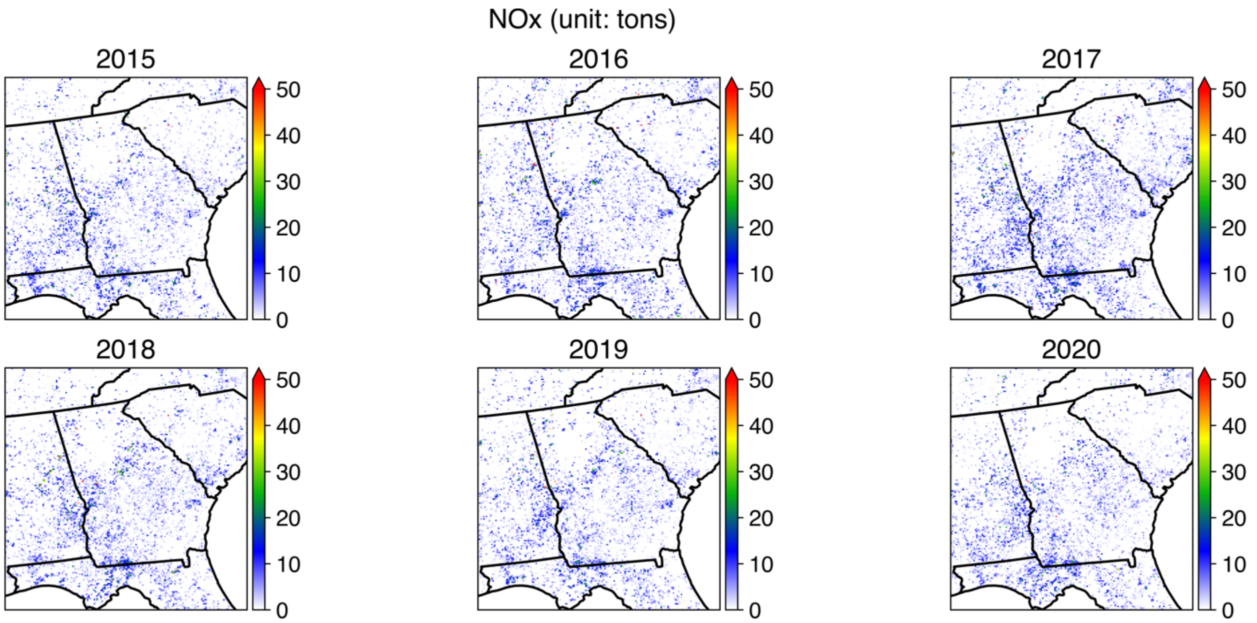

Fig S6. Spatial distribution of yearly prescribed burn emitted NOx (unit: tons).

<u>Statistical measures for model performance evaluation discussed in this paper.</u>

Mean bias (MB) $=\frac{1}{N}\sum(M_j - O_j)$

Mean error (ME) $=\frac{1}{N}\sum|M_j - O_j|$

Root mean square error (RMSE) $= \sqrt{\frac{1}{N}\sum(M_j - O_j)^2}$

Root mean square error (RMSE) $= \sqrt{\frac{1}{N}\sum[(M_j - \overline{M_j}) - (O_j - \bar{O}_j)]^2}$

Normalized mean bias (NMB) $= \frac{\sum(M_j - O_j)}{\sum O_j} \times 100$

Normalized mean error (NME) $= \frac{\sum|M_j - O_j|}{\sum O_j} \times 100$

Mean normalized bias (MNB) $= \frac{1}{N}\sum\frac{(M_j - O_j)}{O_j} \times 100$

Mean normalized error (MNE) $= \frac{1}{N}\sum\frac{|M_j - O_j|}{O_j} \times 100$

Fractional bias (FB) $= \frac{2}{N}\sum\frac{(M_j - O_j)}{(M_j + O_j)} \times 100$

Fractional error (FE) $== \frac{2}{N}\sum\frac{|M_j - O_j|}{|M_j + O_j|} \times 100$

Note. Subscript j represents the pairing of N observations O and Model predictions M by site and time. Overbars signify means over site and/or time.

Mean bias (MB) (in concentration unit); Mean error (ME) (in concentration unit); Root mean square error (RMSE) (in concentration unit); Centered RMSE (CRMSE) (in concentration unit); Normalized mean bias (NMB) (in %); Normalized mean error (NME) (in %); Mean normalized bias (MNB) (in %); Mean normalized error (MNE) (in %); Fractional bias (FB) (in %); Fractional error (FE) (in %); coefficient of determination (R^2).

Table S2. Yearly prescribed burn area and estimated emitted pollutants.

**Parameter**	**2015**	**2016**	**2017**	**2018**	**2019**	**2020**
Burned Area (acres)	1,991,475	2,278,873	2,735,877	2,202,661	2,014,606	1,988,585
$PM_{2.5}$ (tons)	248,622	290,460	345,070	279,238	256,990	249,446
VOCs (tons)	260,091	306,690	365,522	295,646	269,389	260,092
NOx (tons)	28,288	33,364	39,109	31,867	29,362	28,187

Table S3: CAMQ-DF method performances

	$PM_{2.5}$			MDA8-O_3		
	Total study period	Fire Season (January-April)	No-fire Season (May-September)	Total study period	Fire Season (January-April)	No-fire Season (May-September)
MB	-0.39	-0.38	-0.34	-1.73	-1.51	-2.03
ME	1.63	1.58	1.68	3.03	2.81	3.27
RMSE	3.49	2.28	4.64	4.03	3.67	4.35
CRMSE	3.46	2.25	4.63	3.64	3.35	3.85
NMB	-4.65	-4.75	-3.87	-4.38	-3.64	-5.06
NME	19.40	19.75	19.04	7.65	6.79	8.17
MNB	0.65	2.07	0.18	-2.70	-2.17	-3.30
MNE	21.87	23.25	20.50	7.77	7.26	8.01
FB	-4.13	-3.67	-3.79	-3.32	-2.88	-3.88
FE	20.69	21.13	19.88	7.80	7.09	8.17
R^2	0.55	0.71	0.37	0.91	0.89	0.91

Table S4: CAMQ-DF model performances.

Statistics	Tenfold CV		LOLO CV	
	$PM_{2.5}$	MDA8-O_3	$PM_{2.5}$	MDA8-O_3
	Total study period	Total study period	Total study period	Total study period
MB (in concentration unit)	-0.39	-1.73	-0.39	-1.73
ME (in concentration unit)	1.69	3.13	1.63	3.03
RMSE (in concentration unit)	3.53	4.18	3.47	4.03
CRMSE (in concentration unit)	3.51	3.81	3.45	3.64
NMB (in %)	-4.69	-4.36	-4.65	-4.38
NME (in %)	20.15	7.91	19.38	7.66
MNB (in %)	1.55	-2.55	0.64	-2.69
MNE (in %)	23.45	8.12	21.85	7.77
FB (in %)	-4.19	-3.26	-4.13	-3.32
FE (in %)	21.62	8.09	20.67	7.80
R^2	0.54	0.89	0.55	0.90

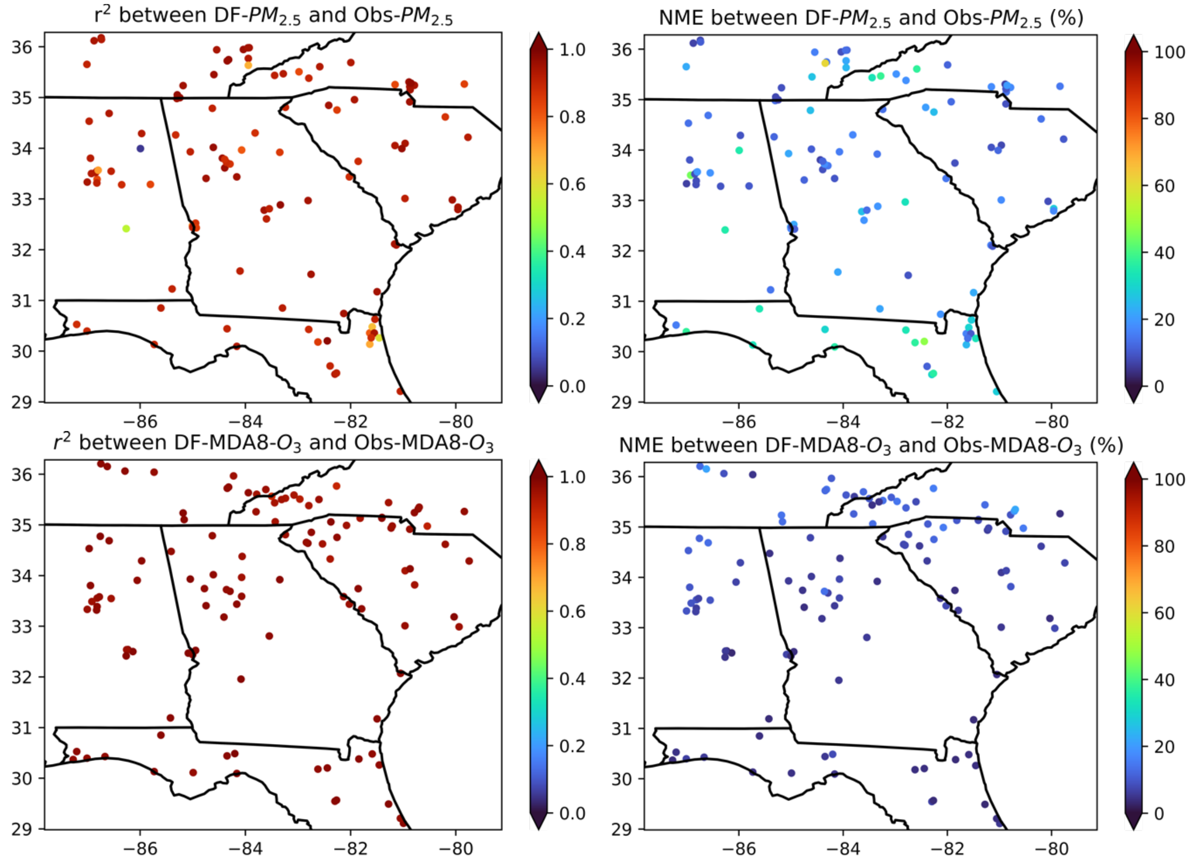

Fig S7. R^2 and normalized mean error (NME) (in %) between CMAQ-DF $PM_{2.5}$ and observed $PM_{2.5}$ (top) and CMAQ-DF MDA8-O_3 and observed MDA8-O_3 (bottom) over the study period.

Table S5: Statistics of the prescribed burn smoke pollution concentrations over the study period

Year	Average over the study domain				Average in Georgia			
	Prescribed $PM_{2.5}$ ($\mu g/m^3$)		Prescribed-MDA8-O_3 (ppb)		Prescribed $PM_{2.5}$ ($\mu g/m^3$)		Prescribed-MDA8-O_3 (ppb)	
	Mean ± SD (MD)	GM, GSD	Mean ± SD (MD)	GM, GSD	Mean ± SD (MD)	GM, GSD	Mean ± SD (MD)	GM, GSD
2015	0.91±1.17 (0.46)	0.37, 5.43	0.30±0.51 (0.11)	0.09, 6.77	1.10±1.57 (0.49)	0.35, 7.70	0.36±0.67 (0.10)	0.08, 10.0
2016	0.96±1.13 (0.56)	0.52, 3.66	0.31±0.54 (0.13)	0.12, 4.79	1.13±1.53 (0.60)	0.52, 4.80	0.38±0.78 (0.13)	0.12, 6.29
2017	1.28±2.10 (0.40)	0.40, 5.61	0.52±0.87 (0.17)	0.16, 5.88	1.39±1.94 (0.65)	0.54, 5.44	0.51±0.96 (0.14)	0.11, 10.6
2018	0.90±1.62 (0.31)	0.26, 6.34	0.37±0.68 (0.14)	0.11, 6.32	1.03±1.65 (0.42)	0.32, 7.01	0.35±0.75 (0.10)	0.07, 11.2
2019	0.80±1.29 (0.36)	0.31, 4.85	0.35±0.53 (0.19)	0.15, 4.86	0.94±1.30 (0.55)	0.38, 5.36	0.32±0.58 (0.15)	0.10, 6.85
2020	0.81±1.18 (0.34)	0.33, 4.31	0.32±0.43 (0.14)	0.14, 4.31	0.93±1.20 (0.51)	0.40, 4.77	0.27±0.44 (0.11)	0.08, 8.56
2015-2020	0.94±1.45 (0.41)	0.35, 5.06	0.36±0.61 (0.15)	0.13, 5.48	1.08±1.54 (0.53)	0.41, 5.85	0.36±0.71 (0.12)	0.09, 8.85

Table S6: Statistics of prescribed burn smoke pollution concentrations over the burning season (January-April)

Year	Average over the study domain				Average in Georgia			
	Prescribed $PM_{2.5}$ ($\mu g/m^3$)		Prescribed MDA8-O_3 (ppb)		Prescribed $PM_{2.5}$ ($\mu g/m^3$)		Prescribed MDA8-O_3 (ppb)	
	Mean ± SD (MD)	GM, GSD	Mean ± SD (MD)	GM, GSD	Mean ± SD (MD)	GM, GSD	Mean ± SD (MD)	GM, GSD
2015	1.19±1.49 (0.53)	0.37, 7.59	0.50±0.71 (0.16)	0.15, 7.42	1.45±2.10 (0.51)	0.38, 10.0	0.62±0.94 (0.18)	0.13, 11.1
2016	1.36±1.65 (0.75)	0.54, 6.00	0.59±0.84 (0.23)	0.18, 8.94	1.72±2.26 (0.86)	0.51, 9.61	0.76±1.23 (0.21)	0.15, 13.8
2017	2.22±2.72 (1.27)	0.88, 5.74	0.95±1.19 (0.56)	0.36, 6.68	2.40±2.57 (1.82)	1.06, 5.86	1.00±1.35 (0.52)	0.24, 16.2
2018	1.73±2.38 (0.79)	0.58, 6.08	0.74±1.01 (0.32)	0.25, 6.16	1.88±2.45 (0.95)	0.61, 6.58	0.75±1.14 (0.27)	0.16, 11.2
2019	1.11±1.68 (0.55)	0.33, 6.89	0.53±0.79 (0.24)	0.15, 8.02	1.25±1.72 (0.66)	0.35, 8.71	0.54±0.89 (0.26)	0.10, 14.0
2020	0.95±1.18 (0.53)	0.42, 4.40	0.43±0.51 (0.24)	0.18, 5.40	1.10±1.29 (0.61)	0.44, 5.65	0.40±0.54 (0.17)	0.11, 11.4
2015-2020	1.43±1.97 (0.67)	0.49, 6.12	0.62±0.88 (0.28)	0.20, 7.16	1.64±2.14 (0.77)	0.52, 7.80	0.67±1.06 (0.26)	0.14, 13.0

Table S7: Statistics of the prescribed burn smoke pollution concentrations over the cold season (October-December)

Year	Average over the study domain				Average in Georgia			
	Prescribed $PM_{2.5}$ ($\mu g/m^3$)		Prescribed-MDA8-O_3 (ppb)		Prescribed $PM_{2.5}$ ($\mu g/m^3$)		Prescribed-MDA8-O_3 (ppb)	
	Mean ± SD (MD)	GM, GSD	Mean ± SD (MD)	GM, GSD	Mean ± SD (MD)	GM, GSD	Mean ± SD (MD)	GM, GSD
2015	1.09±1.32 (0.55)	0.33, 8.45	0.34±0.50 (0.14)	0.07, 14.3	1.22±1.61 (0.52)	0.25, 15.7	0.39±0.62 (0.16)	0.05, 26.4
2016	1.07±0.73 (0.92)	0.80, 2.33	0.26±0.16 (0.26)	0.18, 2.95	1.11±0.91 (0.79)	0.74, 2.73	0.26±0.22 (0.20)	0.15, 4.11
2017	1.39±1.91 (0.71)	0.50, 5.97	0.46±0.62 (0.26)	0.16, 6.06	1.30±1.44 (0.78)	0.53, 5.86	0.41±0.58 (0.19)	0.11, 9.00
2018	0.76±1.15 (0.29)	0.17, 10.4	0.27±0.43 (0.11)	0.06, 10.3	0.74±1.03 (0.25)	0.15, 13.1	0.24±0.40 (0.07)	0.03, 22.9
2019	1.01±1.51 (0.49)	0.38, 5.22	0.31±0.41 (0.16)	0.12, 6.00	1.03±1.44 (0.46)	0.36, 6.04	0.30±0.45 (0.12)	0.09, 7.45
2020	1.50±1.68 (0.78)	0.60, 5.20	0.46±0.53 (0.27)	0.19, 5.00	1.38±1.59 (0.77)	0.58, 5.85	0.40±0.56 (0.15)	0.10, 11.4
2015-2020	1.13±1.46 (0.58)	0.40, 6.57	0.35±0.47 (0.17)	0.12, 7.53	1.12±1.38 (0.60)	0.37, 8.33	0.33±0.50 (0.14)	0.08, 13.1

Table S8: Statistics of the prescribed burn smoke pollution concentration over the no-fire season (May-September)

Year	Average over the study domain				Average in Georgia			
	Prescribed $PM_{2.5}$ ($\mu g/m^3$)		Prescribed-MDA8-O_3 (ppb)		Prescribed $PM_{2.5}$ ($\mu g/m^3$)		Prescribed-MDA8-O_3 (ppb)	
	Mean ± SD (MD)	GM, GSD	Mean ± SD (MD)	GM, GSD	Mean ± SD (MD)	GM, GSD	Mean ± SD (MD)	GM, GSD
2015	0.57±0.46 (0.43)	0.40, 2.42	0.12±0.11 (0.08)	0.08, 2.73	0.64±0.58 (0.44)	0.41, 2.81	0.11±0.10 (0.08)	0.07, 3.14
2016	0.61±0.54 (0.46	0.43, 2.53	0.13±0.12 (0.09)	0.08, 2.73	0.71±0.74 (0.50)	0.45. 2.81	0.14±0.15 (0.10)	0.09, 3.05
2017	0.29±0.35 (0.19)	0.17, 3.22	0.12±0.12 (0.08)	0.07, 3.28	0.47±0.43 (0.33)	0.28, 3.55	0.10±0.10 (0.06)	0.05, 4.97
2018	0.31±0.28 (0.21)	0.18, 3.49	0.14±0.12 (0.11)	0.09, 3.36	0.52±0.50 (0.34)	0.29, 3.64	0.11±0.11 (0.08)	0.05, 4.83
2019	0.42±0.42 (0.30)	0.25, 3.21	0.23±0.17 (0.20)	0.18, 2.24	0.65±0.55 (0.51)	0.43, 2.93	0.17±0.13 (0.15)	0.12, 2.72
2020	0.32±0.34 (0.22)	0.19, 3.07	0.15±0.14 (0.11)	0.10, 2.86	0.54±0.63 (0.39)	0.30, 3.40	0.11±0.11 (0.07)	0.05, 5.01
2015-2020	0.42±0.43 (0.29)	0.25, 3.17	0.15±.14 (0.11)	0.10, 2.96	0.59±0.50 (0.42)	0.36, 3.22	0.12±0.10 (0.10)	0.07, 4.04

GM: Geometric Mean ; GSD: Geometric Standard Deviation.

Table S9: Means and standard deviations of total $PM_{2.5}$ ($\mu g/m^3$) and burn impacts at monitoring sites during 2015–2018.

**$PM_{2.5}$**	**2015**	**2016**	**2017**	**2018**	**2019**	**2020**
Observation	8.62±4.31	8.58±5.37	8.47±8.05	8.05±4.00	8.56±4.11	8.13±4.36
CMAQ Fire	5.71±3.91	5.60±3.68	6.03±4.59	5.50±3.83	5.69±3.70	5.50±3.69
BI (CMAQ)	0.84±1.49	0.81±1.37	1.16±2.02	0.78±1.42	0.73±1.33	0.70±1.11
Fusion-CMAQ	8.35±4.05	8.32±4.86	7.98±4.02	7.73±3.89	8.05±4.00	7.62±4.06
BI (FUSED-CMAQ)	0.94±1.32	1.02±1.28	1.18±1.60	0.93±1.40	0.89±1.27	0.83±1.06

Table S10: Means and standard deviations of total MDA8-O_3 concentrations (ppb) and burn impacts at monitoring sites during 2015–2018.

MDA8-O_3	**2015**	**2016**	**2017**	**2018**	**2019**	**2020**
Observation	38.83±11.49	42.45±10.87	39.57±10.80	39.18±11.10	41.16±11.10	36.19±10.10
CMAQ	51.73±10.06	54.39±9.52	52.04±9.34	51.71±10.12	53.74±10.43	51.75±9.40
BI (CMAQ)	0.33±0.57	0.33±0.56	0.43±0.81	0.32±0.58	0.31±0.54	0.26±0.37
Fusion-CMAQ	37.24±9.53	40.78±9.06	37.73±9.07	37.52±9.29	39.09±9.17	34.58±8.45
BI (FUSED-CMAQ)	0.27±0.49	0.27±0.48	0.36±0.70	0.25±0.49	0.25±0.47	0.20±0.30

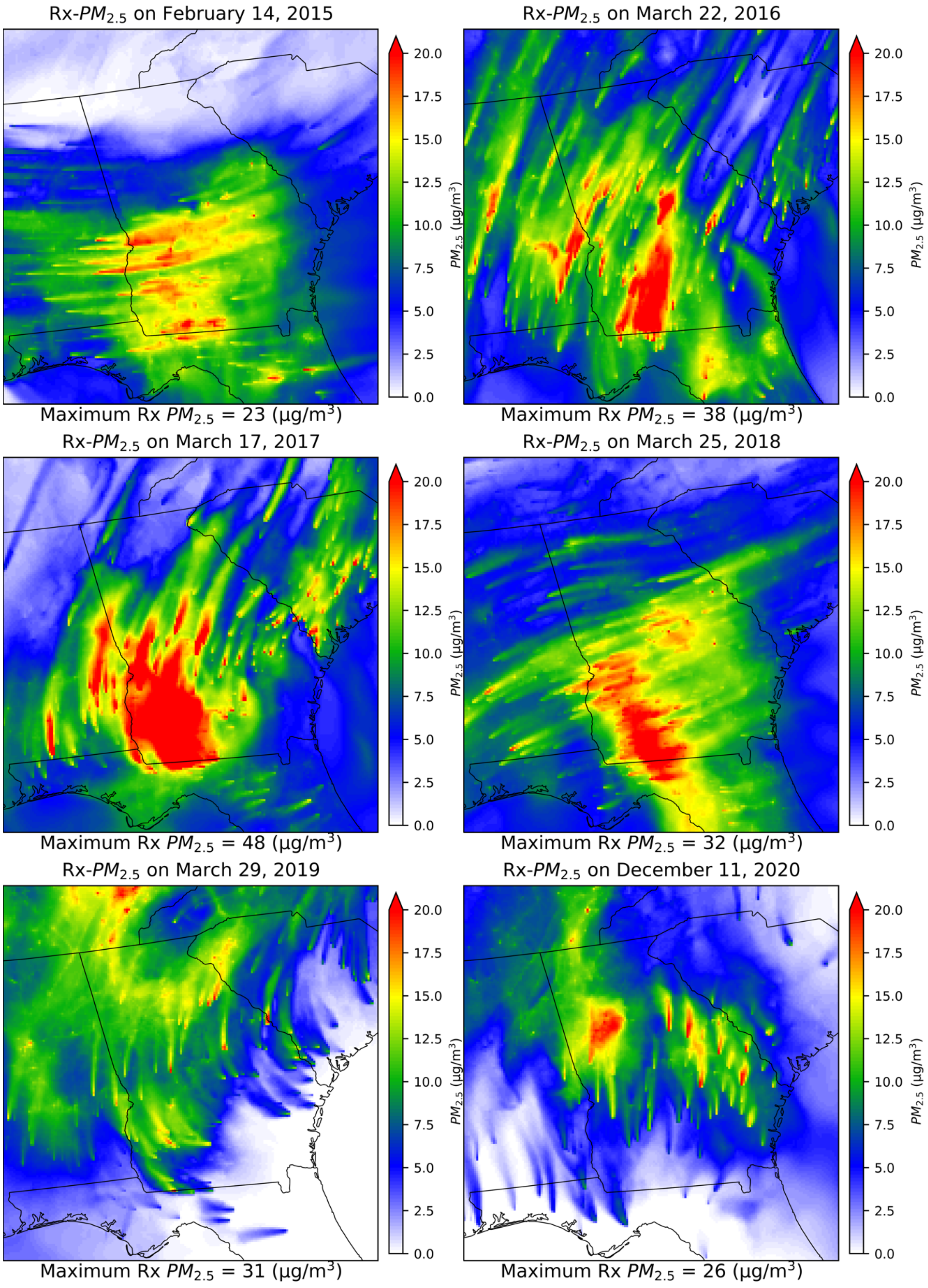

Fig S8. Spatial distribution of daily average prescribed burn smoke $PM_{2.5}$ concentration (μg/m^3) during maximum burned area days.

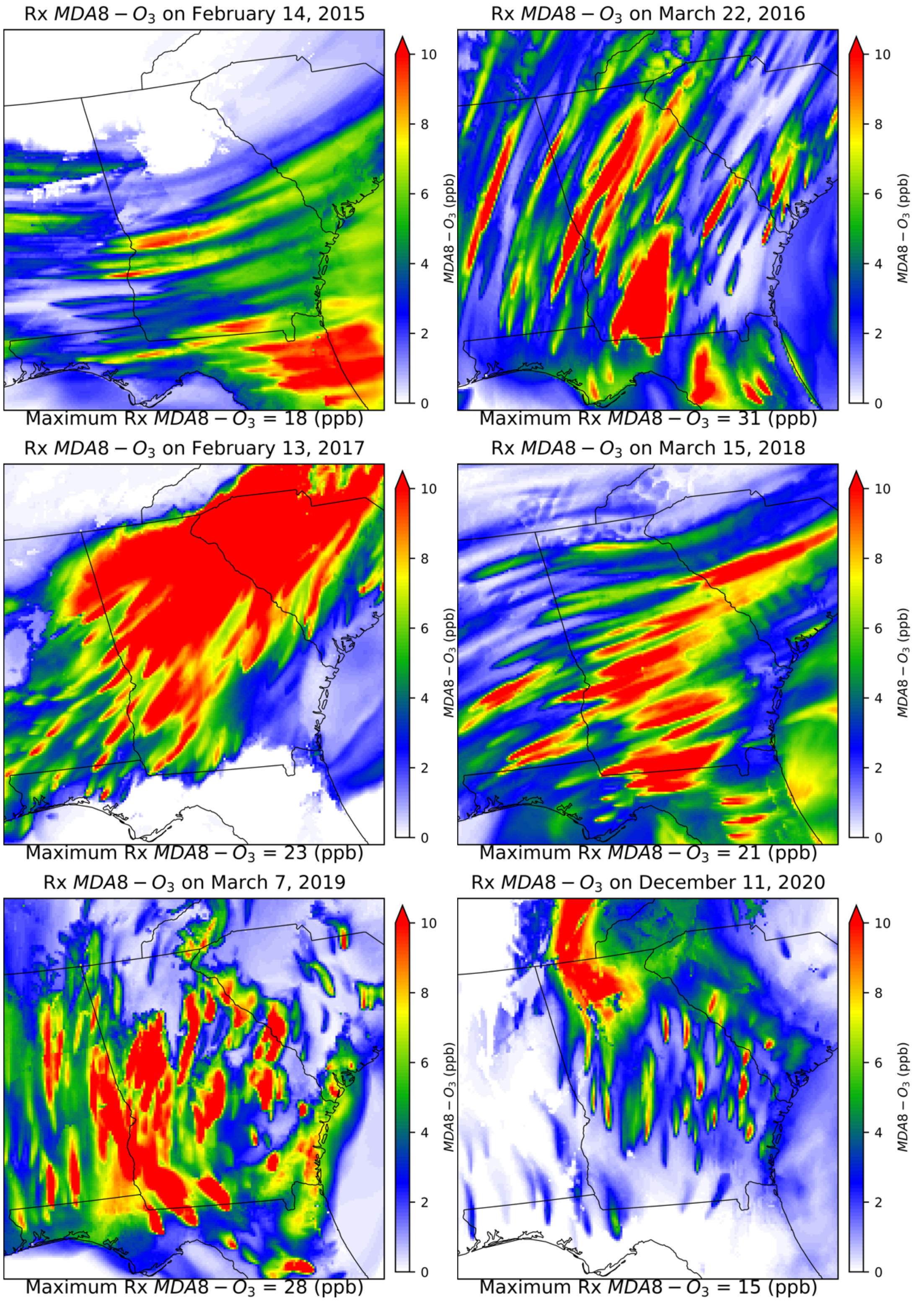

Fig S9. Spatial distribution of daily average prescribed burn smoke MDA8-O_3 concentration (ppb) during maximum burned area days.

Table S11: Yearly average estimated premature deaths over the region attributed to prescribed smoke exposure (grid-level estimation).

Year	Prescribed smoke-$PM_{2.5}$			Prescribed smoke-MDA8-O_3		
	All-cause	Cardiovascular	Respiratory	All-cause	Cardiovascular	Respiratory
2015	447 (377-517)	130 (92-160)	34 (23-45)	35 (22-47)	14 (9-20)	2 (1-3)
2016	488 (412-565)	141 (100-174)	37 (25-49)	38 (24-52)	16 (10-22)	2 (1-3)
2017	646 (545-747)	187 (132-230)	49 (34-64)	64 (40-86)	26 (16-36)	4 (2-5)
2018	463 (391-536)	134 (95-166)	35 (24-46)	46 (29-62)	19 (12-26)	3 (1-4)
2019	445 (375-514)	129 (92-159)	34 (23-45)	48 (30-65)	20 (12-27)	3 (1-4)
2020	426 (359-493)	124 (88-153)	33 (22-43)	40 (26-55)	17 (10-23)	2 (1-3)

Table S12: Yearly average estimated premature deaths in Georgia attributed to prescribed smoke exposure (grid-level estimation).

Year	Prescribed smoke-$PM_{2.5}$			Prescribed smoke-MDA8-O_3		
	All-cause	Cardiovascular	Respiratory	All-cause	Cardiovascular	Respiratory
2015	192 (162-222)	56 (39-69)	15 (10-19)	15 (9-20)	6 (4-8)	1 (0-1)
2016	197 (166-228)	57 (40-70)	15 (10-20)	17 (11-23)	7 (4-10)	1 (0-1)
2017	253 (213-292)	73 (52-90)	19 (13-25)	23 (14-31)	9 (6-13)	1 (1-2)
2018	206 (174-238)	60 (42-74)	16 (11-21)	16 (10-21)	6 (4-9)	1 (0-1)
2019	199 (168-230)	58 (41-71)	15 (10-20)	16 (10-21)	6 (4-9)	1 (0-1)
2020	187 (158-216)	54 (39-67)	14 (10-19)	12 (8-17)	5 (3-7)	1 (0-1)

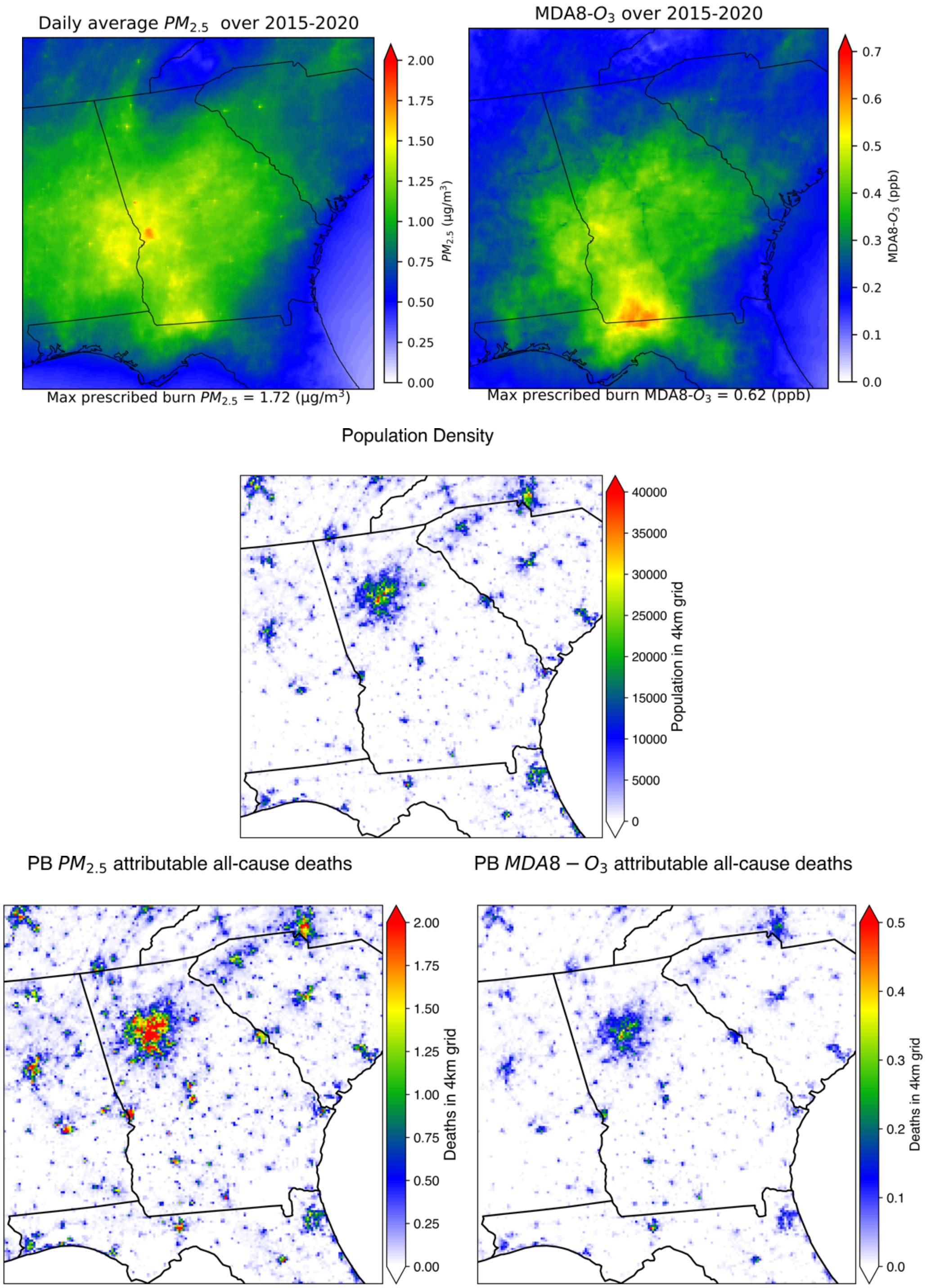

Figure S10. The top figures depict the spatial distribution of daily average $PM_{2.5}$ and MDA8-O_3 resulting from prescribed burns over 2015-2020. The middle figure illustrates the population density across the study region. The bottom figures show the spatial distribution of total all-cause premature deaths attributable to prescribed burn-induced $PM_{2.5}$ and MDA8-O_3 during 2015-2020.

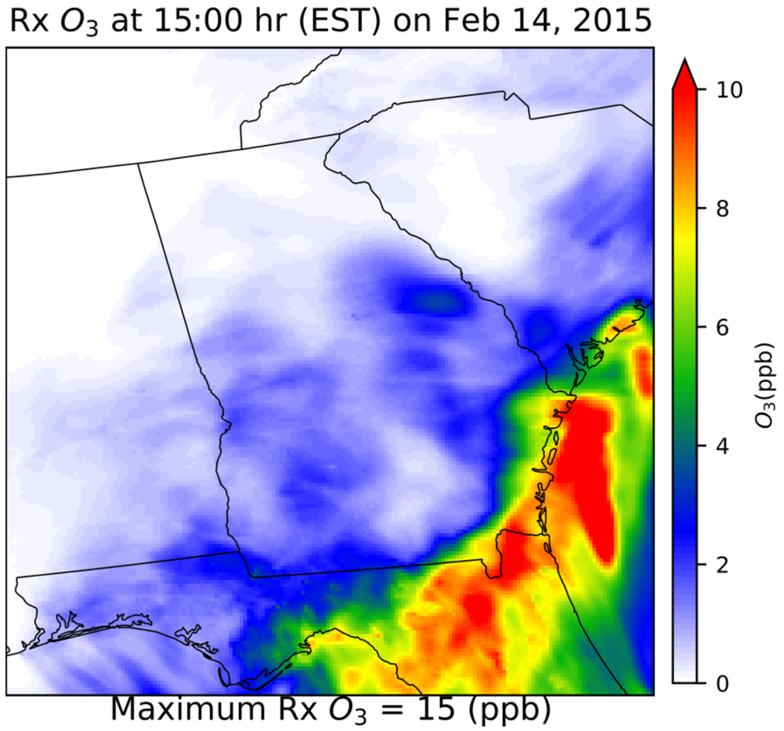

Figure S11. Spatial distribution of prescribed burned O_3 (ppb) at 15:00hr (EST) on Feb 14, 2015. Represent the O_3 formation over the ocean due to prescribed burn smoke.

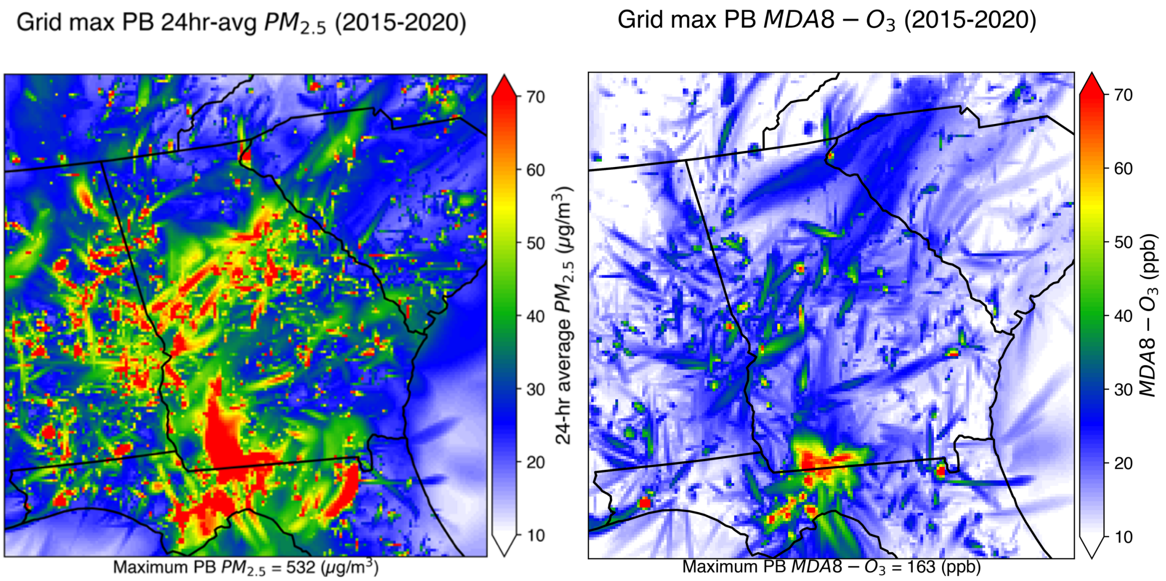

Figure S12. Spatial distribution of gridded maximum 24-hr average $PM_{2.5}$ and MDA8-O_3 during the study period.
